# Expression of NK genes that are not part of the NK cluster in the onychophoran *Euperipatoides rowelli* (Peripatopsidae)

**DOI:** 10.1186/s12861-019-0185-9

**Published:** 2019-04-15

**Authors:** Sandra Treffkorn, Georg Mayer

**Affiliations:** Department of Zoology, Institute of Biology, University of Kassel, Kassel, Germany

**Keywords:** Homeobox genes, NK genes, NK-linked genes, velvet worms, Onychophora, Nephrozoa

## Abstract

**Background:**

NK genes are a group of homeobox transcription factors that are involved in various molecular pathways across bilaterians. They are typically divided into two subgroups, the NK cluster (NKC) and NK-linked genes (NKL). While the NKC genes have been studied in various bilaterians, corresponding data of many NKL genes are missing to date. To further investigate the ancestral roles of NK family genes, we analyzed the expression patterns of NKL genes in the onychophoran *Euperipatoides rowelli*.

**Results:**

The NKL gene complement of *E. rowelli* comprises eight genes, including *BarH*, *Bari*, *Emx*, *Hhex*, *Nedx*, *NK2.1*, *vax* and *NK2.2*, of which only *NK2.2* was studied previously. Our data for the remaining seven NKL genes revealed expression in different structures associated with the developing nervous system in embryos of *E. rowelli*. While *NK2.1* and *vax* are expressed in distinct medial regions of the developing protocerebrum early in development, *BarH*, *Bari*, *Emx*, *Hhex* and *Nedx* are expressed in late developmental stages, after all major structures of the nervous system have been established. Furthermore, *BarH* and *Nedx* are expressed in distinct mesodermal domains in the developing limbs.

**Conclusions:**

Comparison of our expression data to those of other bilaterians revealed similar patterns of *NK2.1*, *vax*, *BarH* and *Emx* in various aspects of neural development, such as the formation of anterior neurosecretory cells mediated by a conserved molecular mechanism including *NK2.1* and *vax*, and the development of the central and peripheral nervous system involving *BarH* and *Emx*. A conserved role in neural development has also been reported from *NK2.2*, suggesting that the NKL genes might have been primarily involved in neural development in the last common ancestor of bilaterians or at least nephrozoans (all bilaterians excluding xenacoelomorphs). The lack of comparative data for many of the remaining NKL genes, including *Bari*, *Hhex* and *Nedx* currently hampers further evolutionary conclusions. Hence, future studies should focus on the expression of these genes in other bilaterians, which would provide a basis for comparative studies and might help to better understand the role of NK genes in the diversification of bilaterians.

**Electronic supplementary material:**

The online version of this article (10.1186/s12861-019-0185-9) contains supplementary material, which is available to authorized users.

## Background

Homeobox genes are a large group of transcription factors that are characterized by the possession of a conserved helix-loop-helix-turn-helix DNA binding motif known as the homeodomain [1–6]. They are involved in various developmental gene-regulatory cascades and typically play a role in morphogenesis, patterning and differentiation processes by binding in a sequence-specific manner to the DNA, mediated by the homeodomain, thus regulating the transcription of specific target genes [1, 4, 5, 7]. Although these genes are found in various metazoans, plants, fungi and even unicellular eukaryotes, bilaterians have evolved the largest diversity of homeobox genes that arose predominantly due to extensive tandem duplications [2, 4, 5]. Based on sequence similarities and phylogenetic analyses, the homeobox genes have been classified into several superclasses, classes, subclasses and families [4, 5, 8, 9]. The homeobox genes of animals are categorized into eleven classes, with the Antennapedia class (ANTP) being one of the largest, comprising the Hox, ParaHox and NK gene families [5, 9].

Extensive studies of the Hox and ParaHox genes in many bilaterians revealed that these genes are involved in molecular pathways that seem to be conserved among bilaterians, including the anterior-posterior patterning system involving the Hox genes and gut patterning mediated by the ParaHox genes (reviewed in ref. [4]). Similar conserved patterns have been observed from other molecular patterning mechanisms such as the determination of the dorsoventral body axis [10, 11], body segmentation [12, 13], or eye development [14–16]. Based on these comparative molecular data, the last common ancestor of all bilaterians (or “urbilaterian”) was proposed to have possessed a complex morphology comprising a segmented body with a centralized nervous system, a differentiated musculature and a vascular system with a pulsatile vessel (reviewed in ref. [14]). However, this hypothesis has been challenged by recent phylogenetic analyses, which have identified the Xenacoelomorpha (Acoela, Nematodermatida and *Xenoturbella*) as the putative sister group of all remaining bilaterians (representatives of Protostomia and Deuterostomia united as Nephrozoa), indicating a rather simple, planula-like morphology of the urbilaterian [14, 17, 18]. Thus, reconstructing the morphology of the urnephrozoan rather than the urbilaterian has become the focus of recent studies.

While the expression patterns and functions of Hox and ParaHox genes have been analyzed in various bilaterians, the third group of ANTP class genes, the NK genes, has only recently become the focus of comparative developmental studies [4, 5, 19]. This might be due to low node support values in phylogenetic analyses, which makes the identification and comparative analysis of NK genes difficult [5, 9]. Interestingly, the sponge *Amphimedon queenslandica* as well as the placozoan *Trichoplax adhaerens* and several ctenophores possess many genes that are similar to the NK genes but lack Hox and ParaHox genes, suggesting that NK genes diversified prior to the emergence of the Hox and ParaHox clusters, most likely due to tandem duplications of a single or several ProtoANTP genes [4, 20–24]. Thus, studies increasingly focus on understanding the evolutionary history of NK genes, which is essential for understanding the evolution of ANTP class homeobox genes and their role in the radiation and diversification of bilaterians [2, 25].

The NK genes, which were first identified in the fruit fly *Drosophila melanogaster* [26], typically comprise two distinct, non-monophyletic groups: the NK cluster (NKC) genes, and the so-called “NK-linked” (NKL) genes [5, 6, 9, 27]. The NKC genes typically comprise one or several clusters within the genomes of many bilaterians [4, 5]. Comparative gene expression and functional studies of the NKC genes in a variety of protostome and deuterostome taxa revealed that these genes are primarily involved in mesoderm and neural patterning [4, 5, 19].

**Fig. 1:**
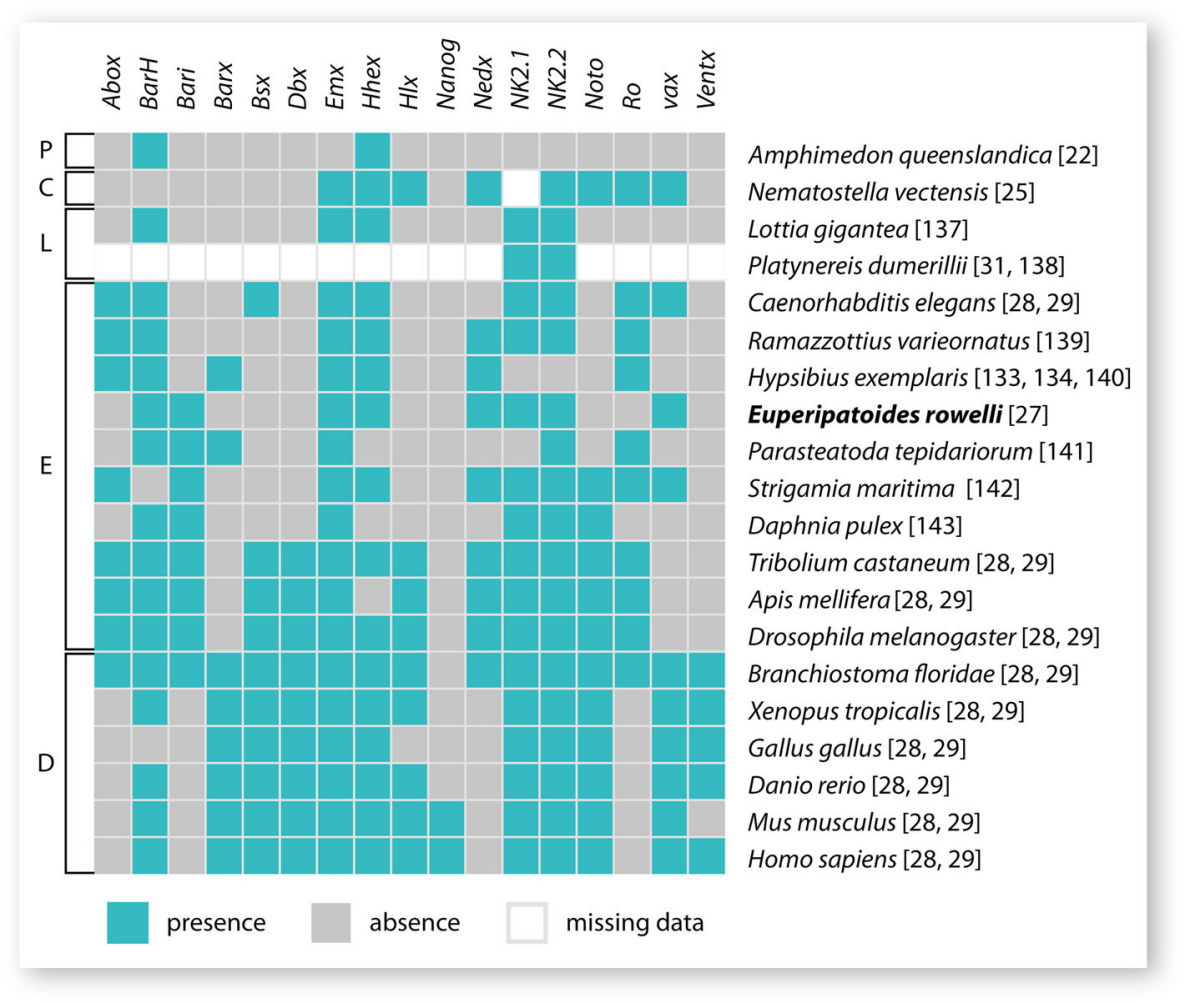
Summary diagram showing NKL gene complements in different metazoan taxa (see also Additional file 1 for further information on the number of NKL genes in different metazoan taxa). Numbers in brackets refer to the corresponding references. Abbreviations: P, Porifera; C, Cnidaria; L, Lophotrochozoa; E, Ecdysozoa; D, Deuterostomia [22, 25, 27–29, 31, 137–143]

In contrast to the NKC genes, the NKL genes lie outside the NK cluster; nevertheless, they have been designated as NK genes because phylogenetic analyses showed that they are more closely related to the NK genes than to any other genes of the ANTP class of homeobox genes [5, 9]. Based on comparative genomic data from various metazoan taxa, a total of 17 NKL genes have been identified, including *Abox*, *BarH*, *Bari*, *Barx*, *Bsx*, *Dbx*, *Emx*, *Hhex*, *Hlx*, *Nanog*, *Nedx*, *NK2.1*, *NK2.2*, *Noto*, *Ro*, *vax* and *Ventx* (Fig. 1) [5, 9, 28, 29]. In contrast to the relatively constant number of NKC genes in most nephrozoans, the number of NKL genes varies to a large degree even within closely related species, which is probably due to extensive gene loss within individual taxa (Fig. 1) [2, 9, 29]. For example, the NKL genes *Abox*, *Bari*, *Nedx* and *Ro* are absent from all vertebrate genomes studied thus far, but orthologs of these genes have been identified in the genome of the cephalochordate *Brachiostoma floridae*, indicating that they were present in the last common ancestor of chordates and were subsequently lost in the lineage leading to vertebrates (Fig. 1) [30]. Furthermore, the gene *Ventx* has so far only been found in chordates, indicating that this gene evolved in the chordate lineage (Fig. 1) [30]. Likewise, *Nanog* has so far only been identified in the mouse and human genomes but its evolutionary origin is still unknown (Fig. 1) [9, 30].

This variability indicates that the NKL genes are more prone to evolutionary changes than the NKC genes, which makes comparative developmental studies difficult. As a result, gene expression data of many NKL genes are still missing to date, in contrast to the well-studied NKC genes [19, 31]. Nevertheless, the genes *BarH*, *Emx*, *NK2.1* and *vax* have been studied in several model species [32–35]. Like the NKC genes, they are involved in many developmental processes that have been used to reconstruct the complex urbilaterian or urnephrozoan [14]. For example, the genes *NK2.1*, *vax*, *Emx* and *BarH* are involved in the development of various neural structures in many nephrozoans, including neurosecretory brain regions, the central and peripheral nervous system, and external sensory structures [33, 35–48]. In addition, *Emx* has been described as a head gap gene in *D. melanogaster*, which is involved in the anterior patterning early in development [14, 49, 50].

In order to get further insights into the evolution of NK homeobox genes among nephrozoans, we analyzed the expression patterns of NKL genes in embryos of the onychophoran *Euperipatoides rowelli*. Together with the Tardigrada and the Arthropoda, the Onychophora (or “velvet worms”) comprise the Panarthropoda, which form the Ecdysozoa (or molting animals) along with the Cycloneuralia ([51, 52]; but see ref. [53] for a critical review of ecdysozoan phylogeny). The NKC and NKL gene complements of *E. rowelli* have been recently characterized [27]. In addition to an almost complete set of NKC genes, transcriptome screenings and phylogenetic analysis revealed a set of eight NKL genes in *E. rowelli*, including *BarH*, *Bari*, *Emx*, *Hhex*, *Nedx*, *NK2.1*, *NK2.2*, and *vax*, while *Abox*, *Barx*, *Bsx*, *Dbx*, *Hlx*, *Nanog*, *Noto*, *Ro* and *Ventx* are missing (Fig. 1) [27]. In this study, we used the previously identified NKL sequences to generate specific probes and conducted *in situ* hybridization on embryos of *E. rowelli*, covering different developmental stages, to clarify the expression patterns of NKL genes during the embryonic development of Onychophora. Together with the expression data of the NKC genes and the NKL gene *NK2.2* of *E. rowelli*, which have been analyzed previously [27], we provide the first expression study of a complete set of NK genes in a panarthropod.

## Results

### Expression of *NK2.1*

**Fig. 2:**
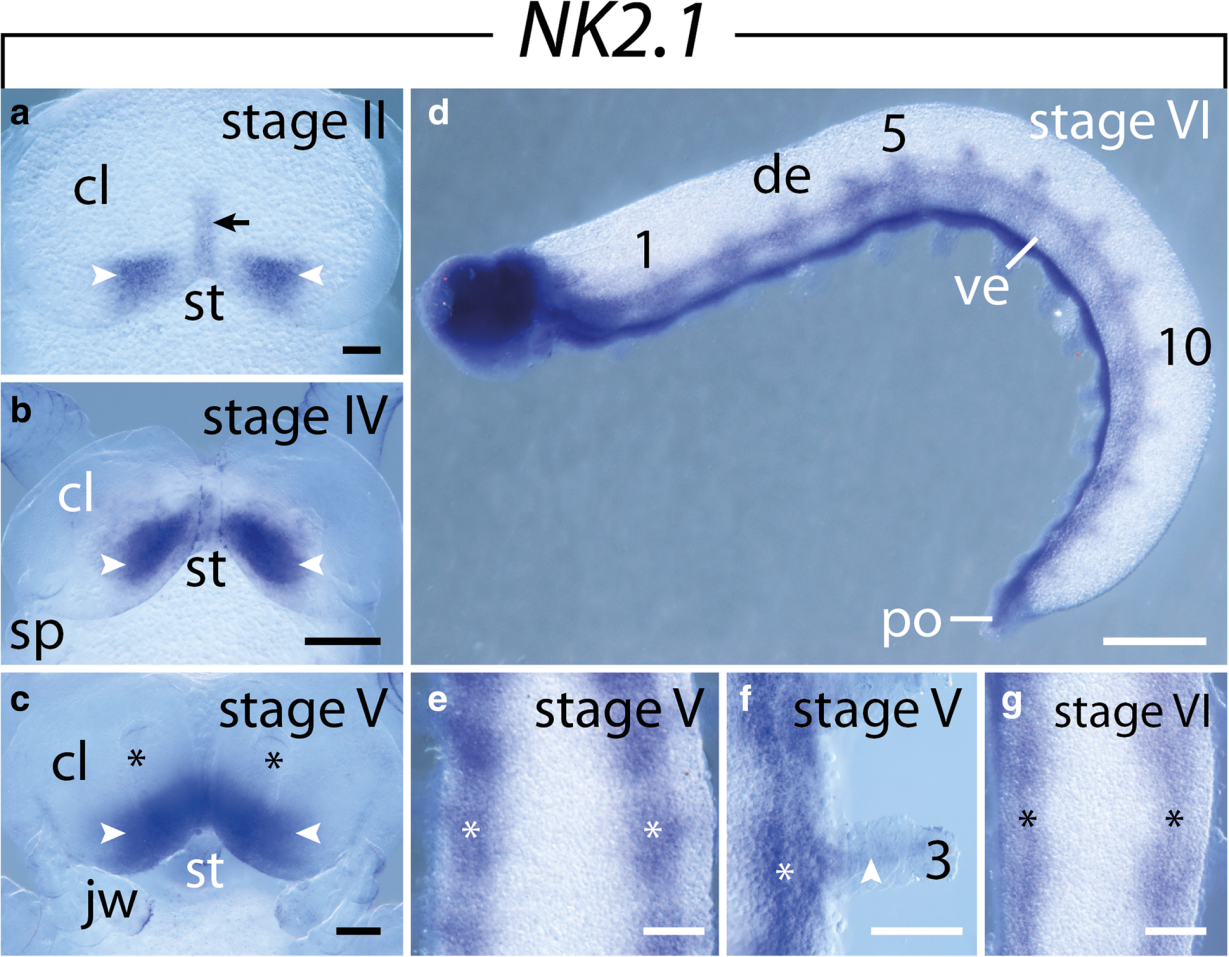
Expression of *NK2.1* at consecutive developmental stages in embryos of the onychophoran *E. rowelli*. Anterior is up in **a**–**c**, **e**–**g** and left in **d**; developing limbs are numbered. **a–c** Expression in the head of consecutive developmental stages in ventral view (arrowheads). Arrow in A indicates an additional domain in the ventral midline of the head, asterisks in C point to the cerebral grooves. **d** Stage VI embryo in lateral view, dorsal is up. **e** Anterior trunk of a stage V embryo in ventral view. Asterisks indicate the expression in the ventral nerve cords. **f** Detail of a developing leg of the same embryo as in E in ventral view. Note the weak expression leading from the nerve cord into the leg (arrowhead). **g** Anterior trunk of a stage V embryo in ventral view. Asterisks indicate the expression in the ventral nerve cords. Abbreviations: cl, cephalic lobe; de, dorsal extra-embryonic tissue; jw, developing jaw; po, proctodeum; sp, developing slime papilla; st, stomodeum; ve, ventral extra-embryonic tissue. Scale bars: **a**, **c**, **g**: 100 μm; **b**, **e**, **f**: 200 μm; **d**: 500 μm

This gene is first expressed in the posterior portion of the cephalic lobes in a narrow medial stripe and a pair of triangular domains lateral to this medial stripe (Fig. 2a). This pattern persists until late in development, when the cerebral grooves (=anlagen of the hypocerebral organs) become visible (Fig. 2b, c). As development proceeds and the cerebral grooves begin to close, *NK2.1* expression is detected in the brain cortex (Fig. 2d). Furthermore, *NK2.1* expression appears along each developing ventral nerve cord, with a stronger signal at the level of the limb buds, and weak signals leading from the nerve cords into each developing limb (Fig. 2e–g).

### Expression of *vax*

**Fig. 3:**
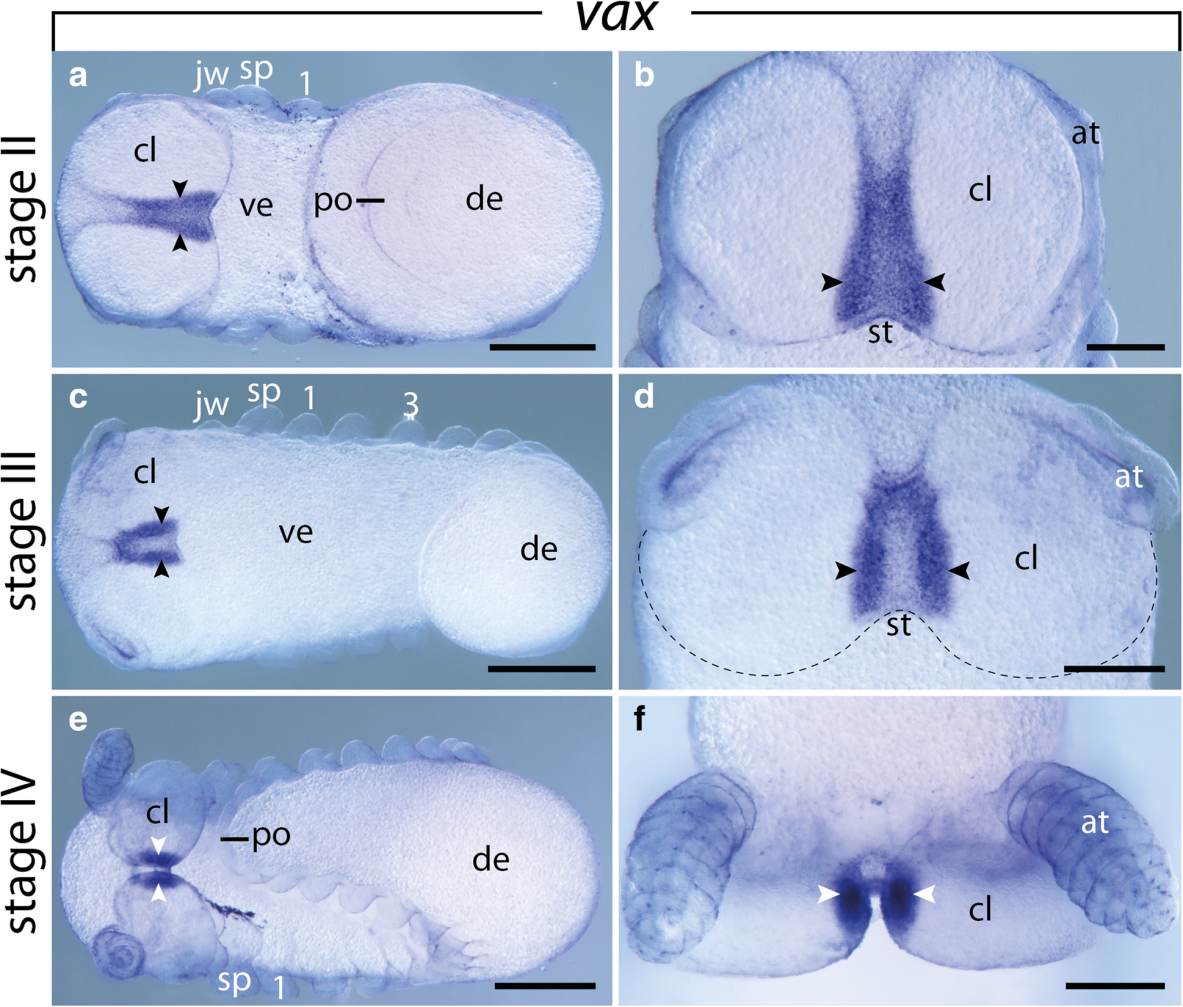
Expression of *vax* at early developmental stages in embryos of the onychophoran *E. rowelli*. Anterior is left in **a**, **c**, **e** and up in **b**, **d**, **f**; developing limbs are numbered. Arrowheads point to the ventromedial expression in the cephalic lobes. **a** Stage II embryo in ventral view. **b** Head of the same embryo as in A in ventral view. **c** Stage III embryo in ventral view. **d** Head of the same embryo as in C in ventral view. Dashed line indicates the posterior border of the cephalic lobes. **e** Stage IV embryo in ventral view. **f** Head of the same embryo as in E in ventral view. Abbreviations: at, developing antenna; cl, cephalic lobe; de, dorsal extra-embryonic tissue; jw, developing jaw; po, proctodeum; sp, developing slime papilla; st, stomodeum; ve, ventral extra-embryonic tissue. Scale bars: **a**, **c**, **e**: 200 μm; **b**, **d**, **f**: 200 μm

**Fig. 4:**
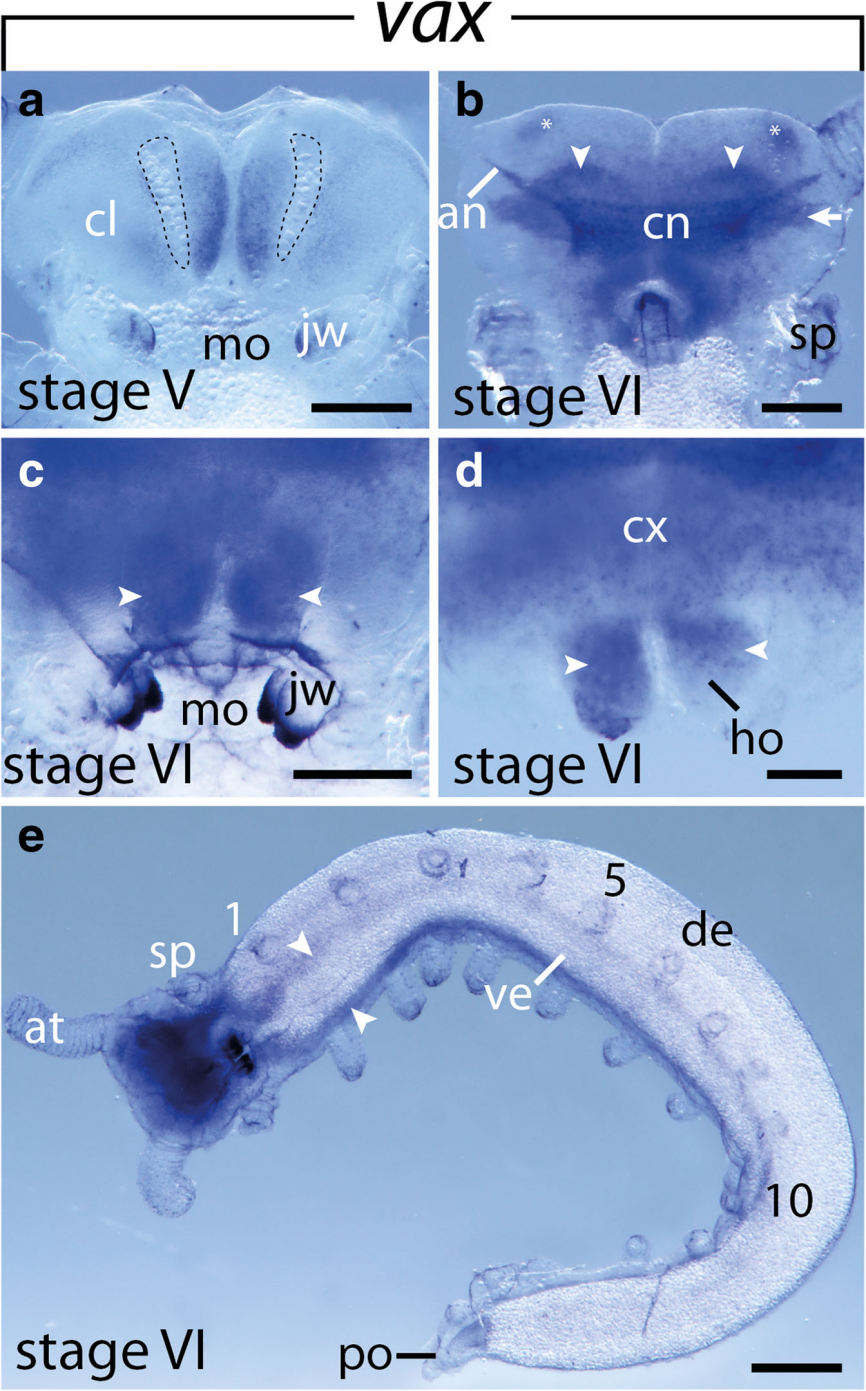
Expression of *vax* at late developmental stages in embryos of the onychophoran *E. rowelli*. Anterior is up in **a**–**c** and left in **e**; developing limbs are numbered. **a** Head of a stage V embryo in ventral view. Dashed lines demarcate the cerebral grooves. **b** Head of a stage VI embryo in dorsal view. Arrows point to uncharacterized regions of the developing brain, arrowheads indicate the putative olfactory lobes. Asterisks indicate the spot-like expression near the base of the antennae. **c** Head of a stage VI embryo in ventral view. Arrowheads point to the expression in the developing hypocerebral organs. **d** Cross section of the head of a stage VI embryo, dorsal is up. Arrowheads point to the expression in the developing hypocerebral organs. **e** Stage VI embryo in ventrolateral view, dorsal is up. Arrowheads point to the expression in the developing ventral nerve cords. Abbreviations: an, antennal tract; at, developing antenna; cl, cephalic lobe; cn, central brain neuropil; cx, brain cortex; de, dorsal extra-embryonic tissue; ho, developing hypocerebral organs; jw, developing jaw; mo, mouth opening; po, proctodeum; sp, developing slime papilla; ve, ventral extra-embryonic tissue. Scale bars: **a**–**d**:100 μm; **e**: 500 μm

The initial expression of *vax* is restricted to a narrow domain in the ventromedial ectoderm along the inner borders of the cephalic lobes anterior to the stomodeum (Fig. 3a, b). This pattern persists up to stage IV (Fig. 3a–f). At stage V, *vax* is still expressed medially of each cerebral groove and its expression persists in the anlagen of the hypocerebral organs after their detachment from the ectoderm and association with the protocerebrum (Fig. 4a–d). Additional *vax* expression appears in the developing brain, including the central brain neuropil, brain cortex and antennal tracts, as well as in each developing ventral nerve cord (Fig. 4b, e).

### Expression of *Emx*

**Fig. 5:**
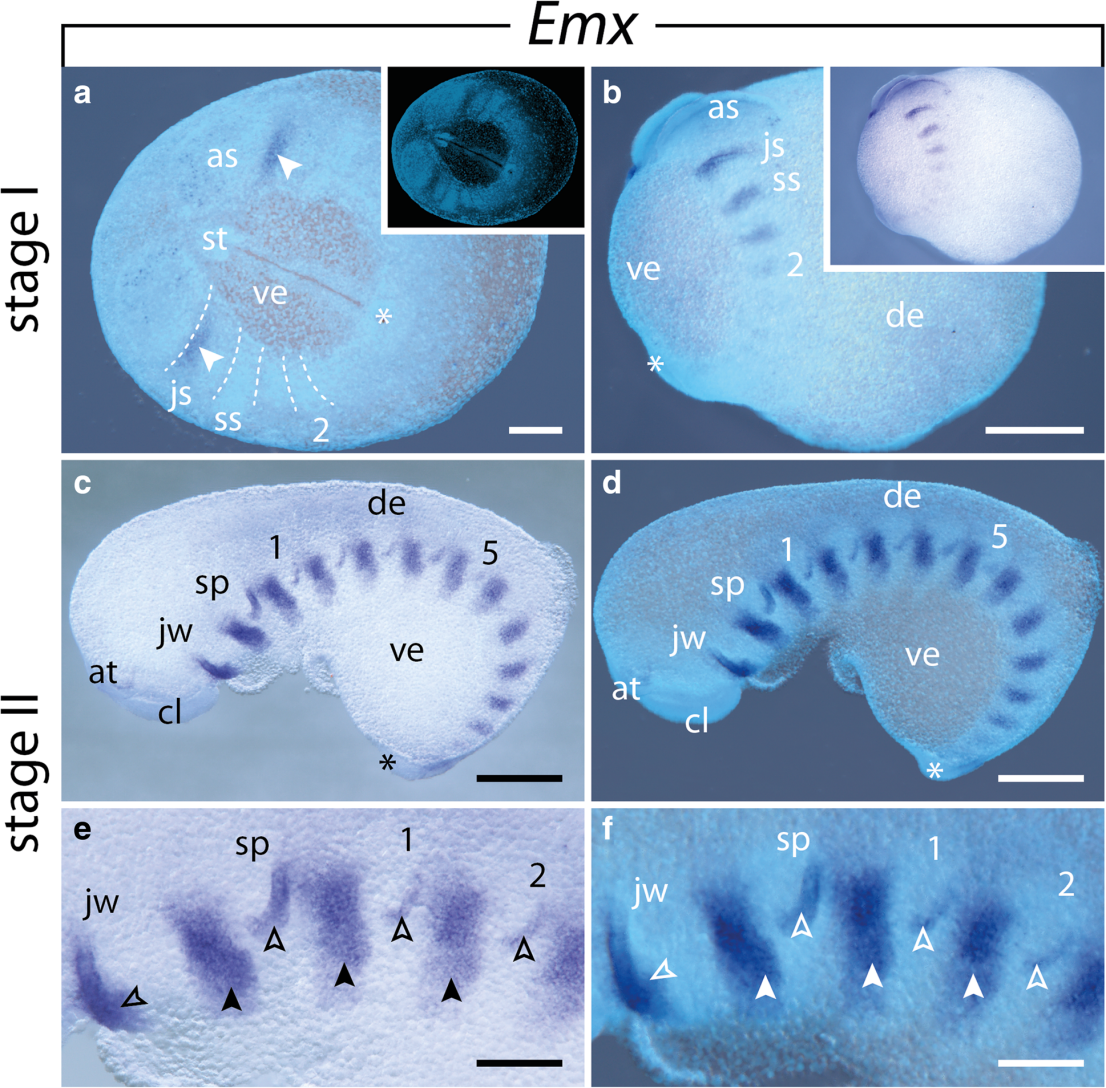
Expression of *Emx* at early developmental stages in embryos of the onychophoran *E. rowelli*. Anterior is left in **a**, **c**–**f** and up in **b**; asterisks demarcate the proctodeum, developing limbs are numbered. **a** Early stage I embryo in ventral view, superimposed light micrograph and DAPI image. Dashed lines indicate the segmental borders, arrowheads point to the expression at the anterior border of the jaw segments. Inset shows the same embryo stained with DAPI. **b** Late stage I embryo in lateral view, superimposed light micrograph and DAPI image, dorsal is right. Inset shows a light micrograph of the same embryo. **c** Stage II embryo in lateral view, dorsal is up. **d** Same embryo as in C, superimposed light micrograph and DAPI image. **e** Anterior developing limbs of a stage II embryo in lateral view, dorsal is up. Filled arrowheads point to the expression at the anterior border of each segment, empty arrowheads indicate the mesodermal expression in the developing limbs. **f** Same detail as in E, superimposed light micrograph and DAPI image. Filled arrowheads point to the expression at the anterior border of each segment, empty arrowheads point to the mesodermal expression in the developing limbs. Abbreviations: as, antennal segment; at, developing antenna; cl, cephalic lobe; de, dorsal extra-embryonic tissue; js, jaw segment; jw, developing jaw; sp, developing slime papilla; ss, slime papilla segment; ve, ventral extra-embryonic tissue Scale bars: **a**, **e**, **f**: 200 μm; **b**–**d**: 500 μm

**Fig. 6:**
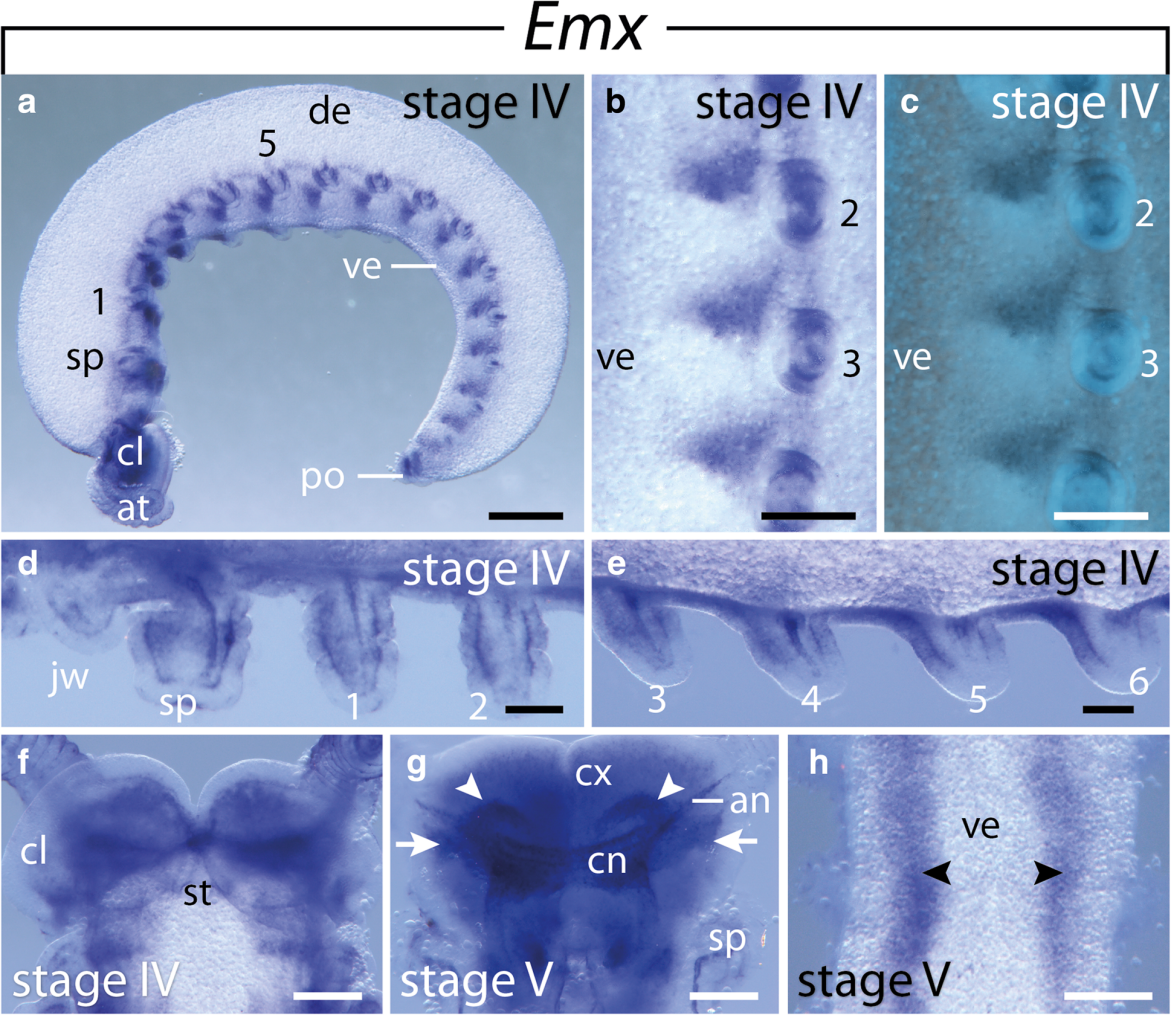
Expression of *Emx* at late developmental stages in embryos of the onychophoran *E. rowelli*. Anterior is left in **a**, **d**, **e** and up in **b**, **c**, **f**–**h**; developing limbs are numbered. **a** Stage IV embryo in lateral view, dorsal is up. **b** Detail of a stage IV embryo in ventrolateral view, dorsal is right. **c** Same detail as in B, superimposed light micrograph and DAPI image. **d** Anterior developing limbs of a stage IV embryo in ventral view. **e** Developing third to sixth legs of the same embryo as in D in ventral view. **f** Head of a stage IV embryo in ventral view. **g** Head of a stage V embryo in dorsal view. Arrows point to uncharacterized regions of the developing brain, arrowheads indicate the putative olfactory lobes. **h** Anterior trunk of a stage V embryo in ventral view. Arrowheads indicate the expression in the developing ventral nerve cords. Abbreviations: an, antennal tracts; at, developing antenna; cl, cephalic lobe; cn, central brain neuropil; cx, brain cortex; de, dorsal extra-embryonic tissue; jw, developing jaw; po, proctodeum; sp, developing slime papilla; st, stomodeum; ve, ventral extra-embryonic tissue Scale bars: **a**: 500 μm; **b**, **c**, **f**–**h**: 200 μm; **d**, **e**: 100 μm

Expression of *Emx* is initiated at early germ band stages before the limbs start to protrude distally from the lateral germ bands (Fig. 5a). A clear stripe appears in the anterior ectoderm of the jaw segment (Fig. 5a). As development proceeds, more *Emx* stripes appear successively and the intensity of the signal increases in the anterior segments, following the anterior-to-posterior progression along the body (Fig. 5b). These domains are restricted to the lateral ectoderm in early stages of development (Fig. 5c–f) and to the ventrolateral ectoderm in later developmental stages (Fig. 6a–c). Additionally, smaller domains appear in the posterior ectoderm of each limb bud except for the antennae and jaws, following the anterior-to-posterior progression of limbs (Fig. 5c–f). As the limb buds protrude distally, these domains extend anteriorly and additional domains appear in the mesoderm of each limb (Fig. 6a–e). In late developmental stages, the segmental ectodermal stripes as well as the expression in the limbs disappear, while a strong expression occurs in the developing brain, including the cortex, central neuropil and antennal tracts, as well as in each developing ventral nerve cord (Fig. 6f–h).

### Expression of *BarH*

**Fig. 7:**
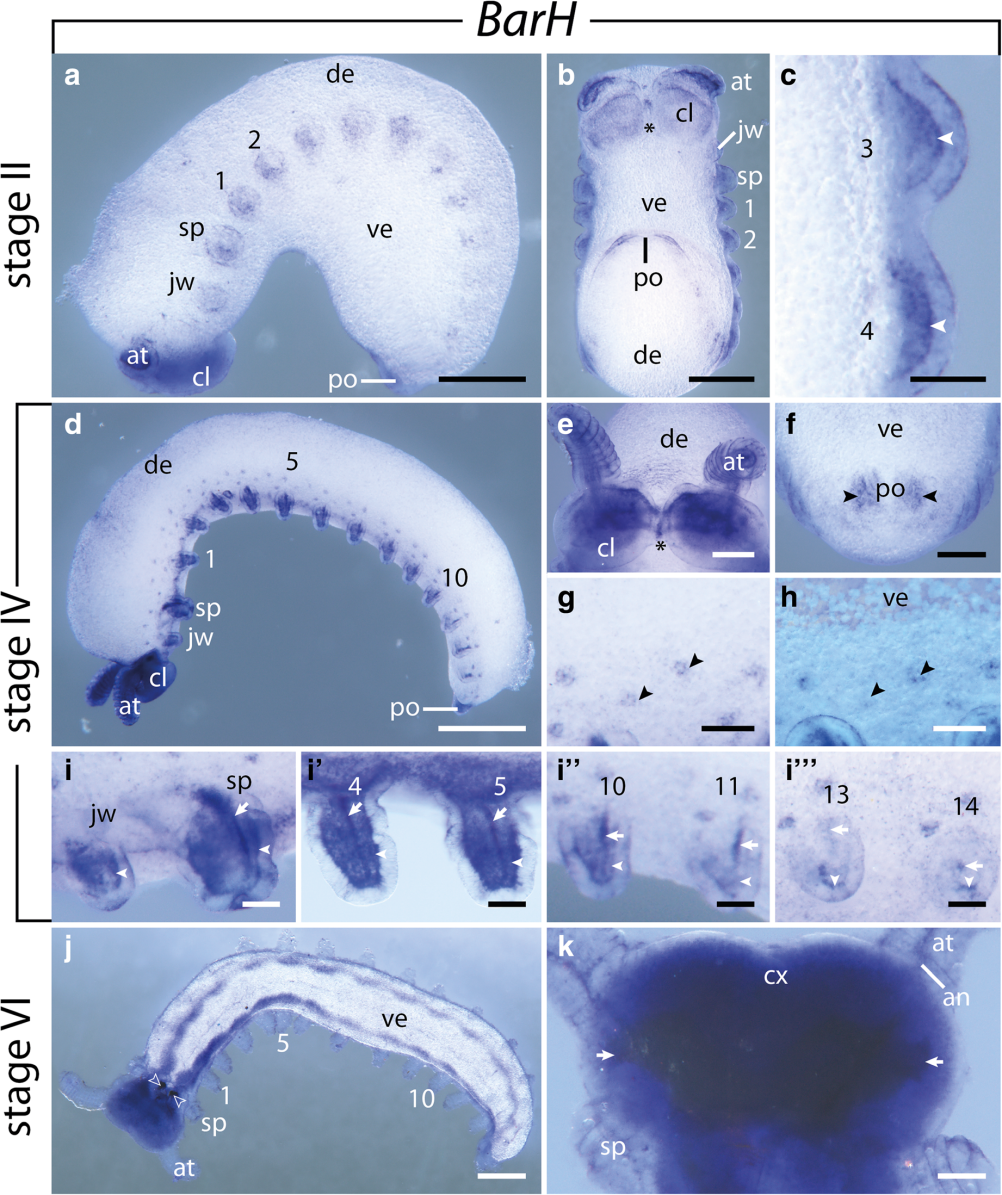
Expression of *BarH* at consecutive developmental stages in embryos of the onychophoran *E. rowelli*. Anterior is left in **a**, **d**, **g**, **h**, **i**–**I**”’, **j** and up in **b**, **c**, **e**, **f**, **k**; asterisks indicate the stomodeum; developing limbs are numbered. **a** Stage II embryo in lateral view, dorsal is up. **b** Same embryo as in A in ventral view. **c** Detail of the third and fouth developing legs in ventral view. Arrowheads indicate the mesodermal expression. **d** Stage IV embryo in lateral view, dorsal is up. **e** Head of a stage IV embryo in ventral view. **f** Posterior end of a stage IV embryo in ventral view. Arrowheads point to the expression around the proctodeum. **g** Detail of the lateral germ band of a stage IV embryo. Arrowheads point to the spot-like expression in the ectoderm dorsal of the developing limbs. **h** Same detail as in G, superimposed light micrograph and DAPI image. Arrowheads point to the spot-like expression in the ectoderm dorsal of the developing limbs. **i–I**”’ Developing limbs of a stage IV embryo in lateral view, dorsal is up. Arrows and arrowheads point to the longitudinal and diffuse mesodermal expression within each limb bud, respectively. **j** Stage VI embryo in ventral view. Empty arrowheads indicate the developing jaws. **k** Head of a stage VI embryo in ventral view. Arrows indicate uncharacterized regions of the brain. Abbreviations: Abbreviations: an, antennal tract; at, developing antenna; cl, cephalic lobe; cx, brain cortex; de, dorsal extra-embryonic tissue; jw, developing jaw; po, proctodeum; sp; developing slime papilla; st, stomodeum; ve, ventral extra-embryonic tissue. Scale bars: **a**, **b**, **d**, **j**: 500 μm; **c**, **g**, **h**: 100 μm; **e**, **f**, **k**: 200 μm; **i**–**I**”’: 50 μm

*BarH* expression is first detectable in stage II embryos in the mesoderm of each limb bud, including the antennae (Fig. 7a–c). A relatively strong expression is also seen in the cephalic lobes (Fig. 7a, b). As the limb buds protrude distally, the mesodermal expression intensifies in the anterior limbs, and a prominent stripe-like domain appears medially in the mesoderm of the anterior limb buds (Fig. 7d, i–I’). While the expression in the cephalic lobes further intensifies, two additional domains appear on either side of the proctodeum (Fig. 7d, f). At the same developmental stage, small spot-like domains appear in two alternating rows along the lateral ectoderm above the limbs (Fig. 7g, h). As development proceeds, the expression in the limbs and the proctodeum as well as the spot-like domains disappear, while the expression in the antennal segment persists and an additional signal appears in the ventral nerve cords (Fig. 7j, k). The latter expression alternates between a strong signal in the limb regions and a weaker expression in the interpedal regions, although the domains in the ventral nerve cords are continuous along the body (Fig. 7j).

### Expression of *Bari*

**Fig. 8:**
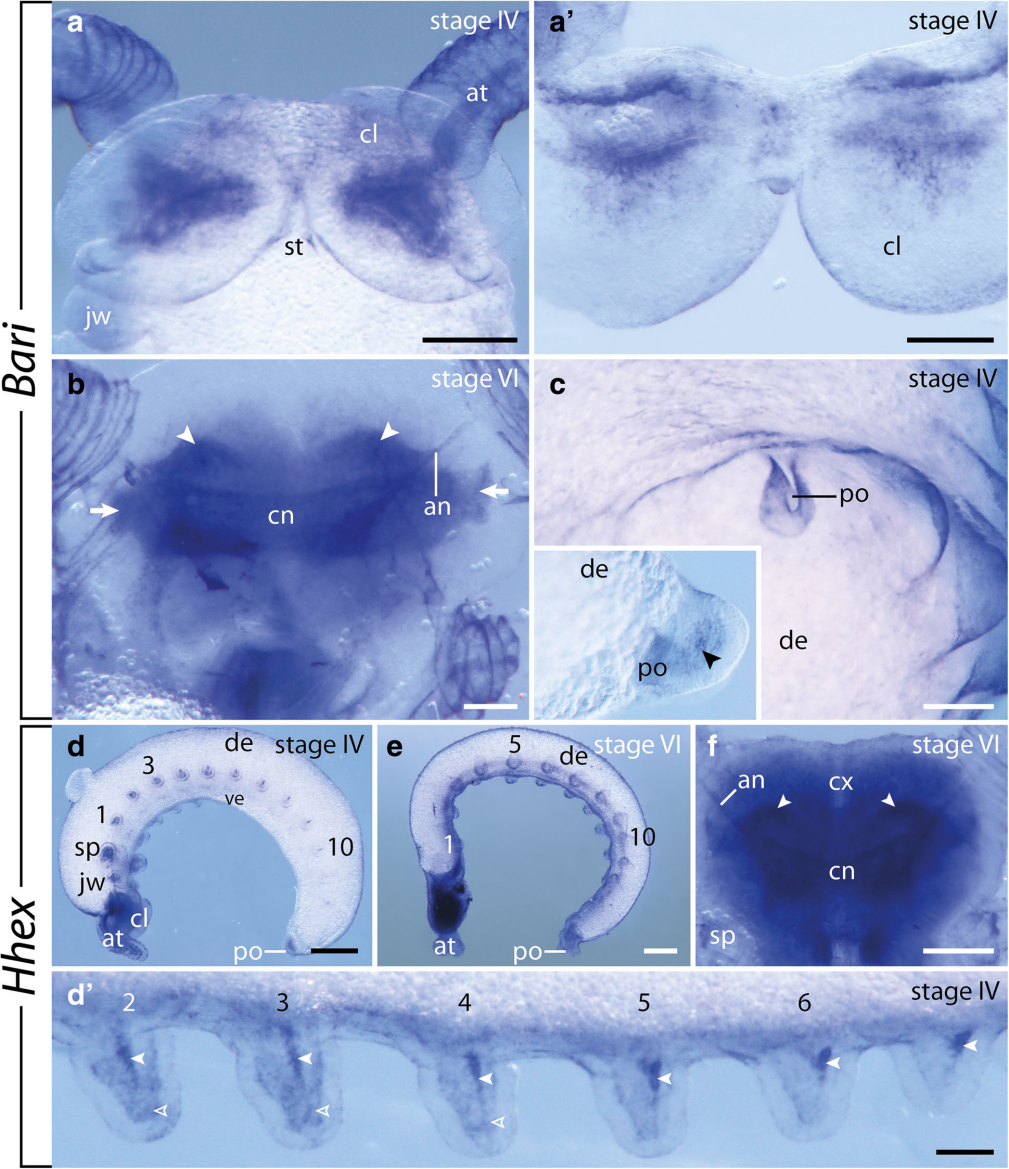
Expression of *Bari* (**a**–**c**) and *Hhex* (**d**–**f**) at late developmental stages in embryos of the onychophoran *E. rowelli*. Anterior is up in **a**, **b**, **c**, **f** and left in **d**, **d**’, **e**; developing limbs are numbered. **a** Head of a stage IV embryo in ventral view. **a**’ Cross section of the head of a stage IV embryo showing the expression in the cephalic lobes. **b** Head of a stage IV embryo in dorsal view. Arrows point to uncharacterized regions of the developing brain, arrowheads indicate the putative olfactory lobes. **c** Posterior end of a stage IV embryo in ventral view. Inset shows the expression in the proctodeum (arrowhead) of the same embryo in lateral view, anterior is left, dorsal is up. **d** Stage IV embryo in lateral view, dorsal is up. **d**’ Anterior developing legs of a stage IV embryo in ventral view. Empty and filled arrowheads point to the diffuse and stripe-like mesodermal domains, respectively. **e** Stage VI embryo in lateral view, dorsal is up. **f** Head of a stage VI embryo in dorsal view. Arrowheads point to the putative olfactory lobes. Abbreviations: an, antennal tract; at, developing antenna; cl, cephalic lobe; cn, central brain neuropil; cx, brain cortex; de, dorsal extra-embryonic tissue; jw, developing jaw; po, proctodeum; sp; developing slime papilla; st, stomodeum; ve, ventral extra-embryonic tissue. Scale bars: **a**, **f**: 200 μm; **b**, **c**, D’: 100 μm; **d**, **e**: 500 μm

The expression of this gene is undetectable until stage IV where it appears as a pair of triangular domains in the cephalic lobes (Fig. 8a, A’). Furthermore, a weak signal is detected around the proctodeum (Fig. 8c). At late developmental stages, the expression appears in the central brain neuropil, antennal tracts, and presumptive olfactory lobes of the developing brain (Fig. 8c).

### Expression of *Hhex*

Expression of *Hhex* was undetectable until stage IV. At this stage, a diffuse expression appears in the mesoderm of each limb bud, in addition to a prominent spot, which elongates following the anterior-to-posterior progression and limb growth (Fig. 8d, D’). As development proceeds, the signal disappears from the developing limbs, whereas a strong expression appears in the developing brain and ventral nerve cords (Fig. 8e, f).

### Expression of *Nedx*

**Fig. 9:**
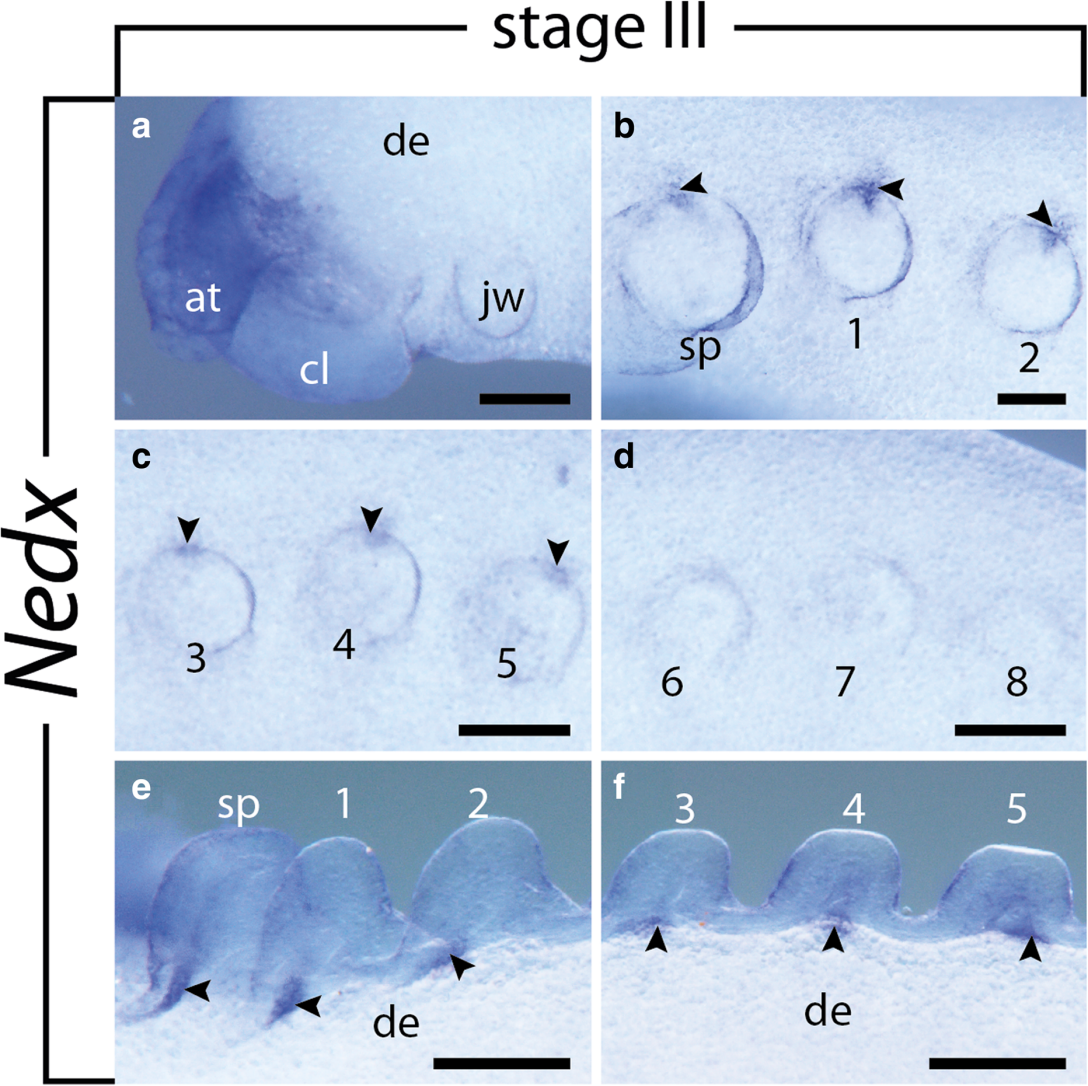
Expression of *Nedx* at stage III embryos of the onychophoran *E. rowelli*. Anterior is left in all images; developing limbs are numbered. Arrowheads point to the spot-like expression dorsal of the developing limbs. **a** Head in lateral view, dorsal is up. **b–d** Consecutive developing limbs in lateral view, dorsal is up. **e–f** Consecutive developing limbs in dorsal view. Abbreviations: at, developing antenna; cl, cephalic lobe; de, dorsal extra-embryonic tissue; jw, developing jaw; sp; developing slime papilla. Scale bars: 200 μm

**Fig. 10:**
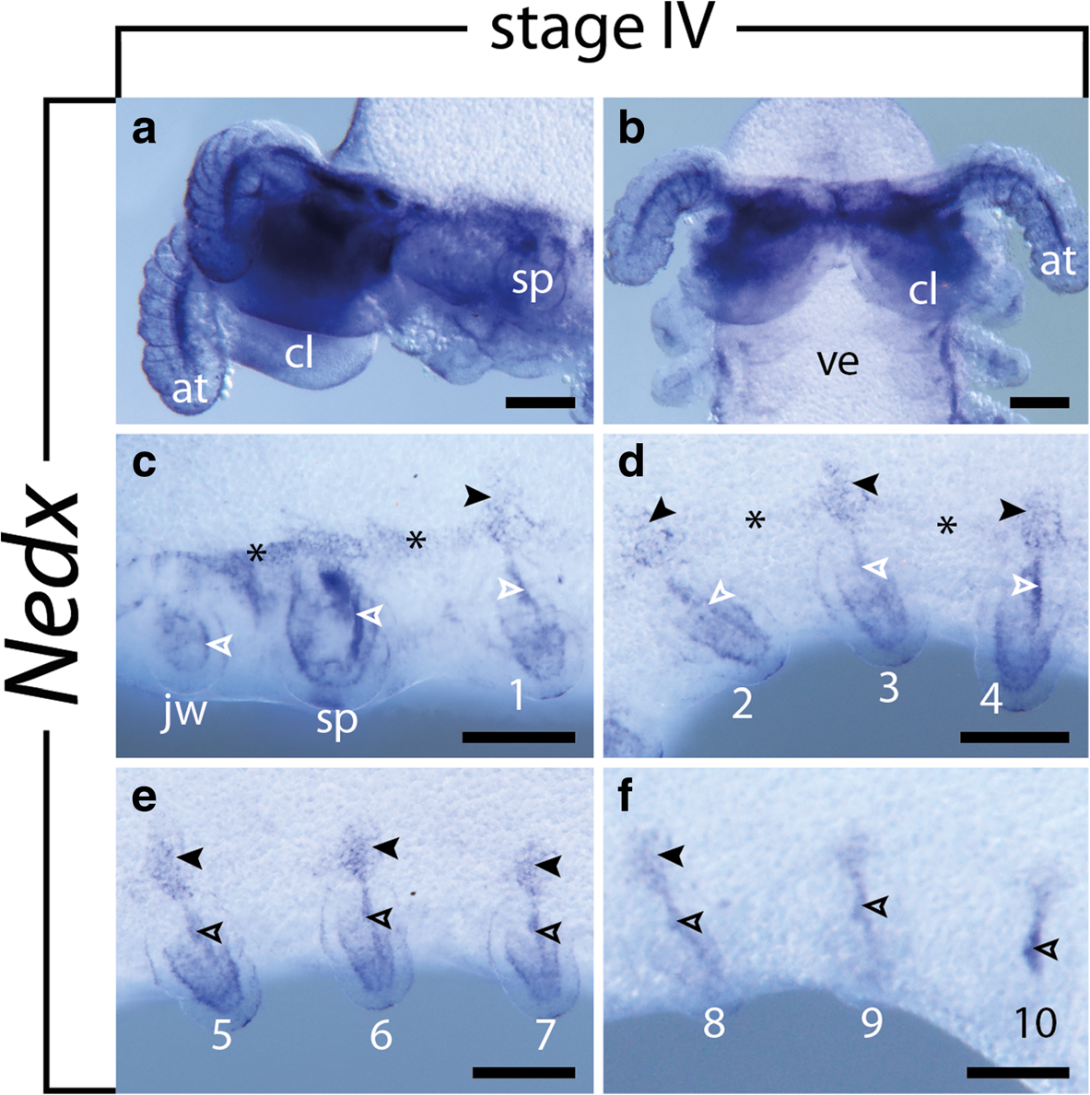
Expression of *Nedx* at stage IV embryos of the onychophoran *E. rowelli*. Anterior is left in **a**, **c**–**f** and up in **b**; developing limbs are numbered. Filled and empty arrowheads point to the spot-like expression dorsal of the developing limbs and the longitudinal mesodermal domains within the limbs, respectively. Asterisks indicate the longitudinal band of expression connecting the spot-like domains. **a** Head in ventrolateral view, dorsal is up. **b** Head in ventral view. **c–f** Consecutive developing limbs in lateral view, dorsal is up. Abbreviations: at, developing antenna; cl, cephalic lobe; jw, developing jaw; sp; developing slime papilla; ve, ventral extra-embryonic tissue. Scale bars: 200 μm

The expression of *Nedx* is first detectable in stage III embryos as prominent domains in the developing brain, including the cortex, the central neuropil, and the antennal tracts, which persists until stage IV (Figs. 9a, 10a, b, Additional file 2). Additional spot-like mesodermal domains occur at the dorsal base of the slime papillae and the first five legs (Fig. 9b–f, Additional file 2A). As development proceeds, more segments express *Nedx* in a similar pattern, while the domains change their shape in the anterior, more developed segments (Fig. 10c–f, Additional file 2B, C). The initially spot-like domains elongate and expand into the distal portion of each limb, following limb growth (Fig. 10c–e, Additional file 2B, C); on the other hand, they show a dorsal expansion, as they are transformed into transverse, dorsoventral stripes on each side of the embryo (Fig. 10c–e, Additional file 2B, C). Moreover, they become interconnected by an additional, continuous, longitudinal domain along each side of the embryo (Fig. 10c, d, Additional file 2B, C).

## Discussion

### Conserved and derived aspects of NKL gene expression in the central and peripheral nervous system of the onychophoran *E. rowelli*

NKL genes show distinct patterns of expression in the developing nervous system in embryos of *E. rowelli*. While *NK2.1*, *vax* and *BarH* are expressed from early stages onwards and show dynamic expression patterns in the developing nervous system until late in development, neural expression of *Hhex*, *Nedx*, *Bari* and *Emx* is initiated later, when all major structures of the nervous system have been established and the developing nerve cords have delaminated from the remaining ventral ectoderm [54–57]. This indicates that *NK2.1* and *vax* are involved in early aspects of neurogenesis (i.e. the formation of neurons from neural precursors and their segregation from the neuroectoderm [58, 59]) and neural development (i.e. the differentiation of neurons, including the formation of axons, dendrites and synapses [59, 60]) in onychophorans, while *Hhex*, *Nedx*, *Bari* and *Emx* might be involved in subsequent neural differentiation and specification rather than neurogenesis.

*NK2.1* and *vax* are expressed in distinct, partially overlapping domains in the ventromedial cephalic ectoderm of *E. rowelli*, which gives rise to the medial part of the protocerebrum [54, 61]. While *vax* is restricted to a narrow medial domain, the *NK2.1* domain extends further laterally. In later developmental stages, *vax* expression additionally occurs in the cerebral grooves, the surrounding tissue of which gives rise to the hypocerebral organs that become associated ventrally with the protocerebrum [54, 55, 58, 61–66]. These patterns are reminiscent of what has been described from the centipede *Strigamia maritima*, where *NK2.1* and *vax* are expressed in an anterior medial population of neural precursors, which is distinct from precursors arising from the segmental neuroectoderm [48]. In the centipede, this anterior medial region is characterized by the expression of the anterior patterning genes *six3*, *FoxQ2*, *NK2.1*, *rx*, *hbn* and *vax* and forms an early active neurosecretory center (Table 1) [48].

**Table 1:** Expression profiles of genes involved in the development of presumptive neurosecretory brain centers during development of the onychophorans *Euperipatoides rowelli* and *E. kanangrensis*, the centipedes *Strigamia maritima* [48], and the millipede *Glomeris marginata* [67]

Gene	Onychophora (*Euperipatoides rowelli* & *E. kanangrensis*)	Myriapoda (*Strigamia maritima* & *Glomeris marginata*)
	Medial protocerebrum/ hypocerebral organs	Anterior medial region
*six3*	+	+
*FoxQ2*	+	+
*NK2.1*	+	+
*rx*	+	+
*hbn*	+	+
*vax*	+	+
*vsx*	+	–

Information on the expression of *six3*, *FoxQ2*, *NK2.1*, *rx*, *hbn* and *vsx* from the onychophoran *E. kanangrensis* was obtained from refs [67, 68]

It has been shown previously [67–69] that the same set of anterior patterning genes is expressed in similar, partially overlapping medial domains in the antennal segment of the onychophoran *Eupreipatoides kanangrensis*. Thus, our data support the previous assumption [67] that a similar set of genes is expressed in the medial region of the onychophoran antennal segment and the anterior medial region of *S. maritima* (Table 1). Interestingly, the ventromedial protocerebrum of onychophorans seems to harbor a high number of neurosecretory cells [54, 62]. For example, the majority of neurons expressing pigment-dispersing factor – a neuropeptide involved in processes such as dispersal of visual shading pigment and circadian clock in arthropods [70–74] – is located within the medial protocerebrum of the onychophoran brain [75]. Furthermore, even if the hypocerebral organs do not possess neurosecretory cells, they might receive signals from the neurosecretory cells of adjacent medial brain tissue, thus being functionally equivalent to the *corpora allata* of insects [62, 76, 77]. This is similar to the putative neurosecretory function of the anterior medial region of *S. maritima*, indicating that the complementary expression of *six3*, *FoxQ2*, *NK2.1*, *rx*, *hbn* and *vax* might have a conserved role in the development of neurosecretory brain centers in panarthropods [48]. A similar set of genes, including at least *FoxQ2*, *NK2.1* and *hbn*, is expressed in the head of the pill millipede *Glomeris marginata* [67]. Even though these patterns have so far not been investigated with respect to neural structures or the existence of a distinct anterior medial region in this species, the strikingly similar expression patterns to those in *S. maritima* indicate that these genes might be responsible for the development of neurosecretory brain centers in *G. marginata* as well.

Similar to the situation in onychophorans and myriapods, *NK2.1* is expressed in the anterior medial region of the head in the red flour beetle *Tribolium castaneum*, which develops into the *pars intercerebralis*, the neurosecretory center of the insect brain [33, 78]. However, a *vax* ortholog is absent from all insect genomes studied thus far [33, 47, 48], suggesting that this gene might have been lost in the hexapod lineage. Nevertheless, the similarities between onychophorans and arthropods indicate that the panarthropod ancestor might have possessed an anterior neurosecretory brain center, which was characterized by the complementary expression of the transcription factors *six3*, *FoxQ2*, *NK2.1*, *rx*, *hbn* and *vax*.

Notably, similar expression patterns have been reported from various other protostome and deuterostome taxa, including annelids [33, 47], echinoderms [79], and various vertebrates [33, 69, 80–82], where the corresponding regions give rise to the anterior neurosecretory brain centers, including the apical organ of marine invertebrate larvae and the hypothalamus of vertebrates. Based on the similar expression patterns of *NK2.1*, *vax*, *six3*, *rx*, *hbn* and *FoxQ2*, it has been proposed that the anterior neurosecretory brain centers share a common origin and that the bilaterian ancestor (or at least the nephrozoan ancestor, since data from acoels do not provide evidence for a role of these genes in the development of neurosecretory cells [14]) already possessed an anterior medial population of neurosecretory cells characterized by a similar set of transcription factors [33, 47]. Additionally, Tessmar-Raible [33] put forward the hypothesis that these early neurosecretory systems might have consisted of dual-functional, sensory-neurosecretory cells that combine both sensory and neurosecretory functionality, as these cells are the common minimal regulatory unit of neurosecretory centers of many protostome and deuterostome taxa. For example, photosensory-neurosecretory cells expressing the light-sensitive ciliary opsins have been shown to be present in neurosecretory brain regions in vertebrates and invertebrates [33, 47, 83–85]. Interestingly, previous studies revealed that the ciliary opsin of *E. rowelli* is expressed in the medial protocerebrum, which most likely develops from the embryonic region expressing *NK2.1* and *vax* [86], indicating that dual-functional, sensory-neurosecretory cells might be present in onychophorans as well. Thus, our data as well as previously published expression data [67, 86] support the hypothesis [33, 47] that the bilaterian or at least the nephrozoan ancestor possessed an anterior population of neurosecretory cells, which was patterned by the complementary expression of *NK2.1*, *vax*, *six3*, *rx*, *hbn* and *FoxQ2*, and that these cells displayed combined sensory-neurosecretory functionality.

Similar to *NK2.1* and *vax*, neural expression of *BarH* is initiated early in development in a diffuse pattern in the cephalic lobes that give rise to the protocerebrum [54, 61]. In contrast to this, expression in the developing nerve cords does not appear until late in development, when the major structures of the nervous system have been established [56, 57, 77]. Additional *BarH* expression occurs in distinct spots that are distributed along the ectodermal bands. While the expression in the brain and nerve cords is most-likely correlated to neural development, the spot-like domains do not correspond to any known structure of the central nervous system. Instead, these domains might correspond to clusters of cells that show an anti-HRP immunoreactivity characteristic of peripheral neurons [58]. These cell clusters do not generate neurons of the ventral nerve cords but rather give rise to dermal papillae – external sensory structures that cover the body surface of onychophorans [58]. Similar spot-like expression patterns have been reported from a *Bar* gene (which may or may not be the ortholog of *BarH* from *E. rowelli*), and the genes *Delta* and *rotund* in the onychophoran *E. kangangrensis*, which have been correlated to sensory organ precursors as well [87–89].

This situation is reminiscent of what has been reported from the fruit fly *D. melanogaster*, which possesses two redundant copies of *BarH* including *BarH1* and *BarH2* [42, 43, 90]. During embryogenesis, both copies are expressed relatively late in development in subsets of neurons of the developing brain and ganglia as well as in neurons of external sensory organs [42, 43]. In the larvae, the expression of *BarH1* and *BarH2* regulates the formation of microchaetae in the anterior part of the notum [90]. These striking similarities in timing and position of *BarH* expression between onychophorans and insects indicate a possible conserved function of this gene in the development of sensory and neural structures in panarthropods. Correspondingly, *BarH* expression data from various vertebrates, such as the frog *Xenopus laevis* [45], the zebrafish *Danio rerio* [46] and the mouse *Mus musculus* [44], show similar patterns in the developing central nervous system, including the diencephalon, dorsal thalamus and retinal ganglion cells. This suggests that neural expression of *BarH* is an ancestral feature of bilaterians or at least the nephrozoans, as comparative data are still missing from xenacoelomorphs.

Similar to *BarH*, *Hhex* is expressed in the developing brain of *E. rowelli*, including its cortex and central neuropil, as well as in the developing nerve cords. In contrast to *NK2.1*, *vax* and *BarH*, however, neural expression of *Hhex* is restricted to late developmental stages, indicating a role in neural development and differentiation rather than neurogenesis. To our knowledge, *Hhex* expression has not been examined in any other invertebrate so far, except for some preliminary data from the fruit fly *D. melanogaster* based on high-throughput screening conducted by the Berkeley Drosophila Genome Project [91–94], which revealed a ubiquitous expression in early developmental stages, and a distinct expression in the posterior midgut primordia and the midgut chamber in late developmental stages. In addition, *Hhex* expression has been characterized in various vertebrate models, including the frog *X. laevis* [95], the zebrafish *D. rerio* [96], and the mouse *M. musculus* [97, 98]. In these taxa, *Hhex* is expressed in the anterior visceral endoderm, which is required for normal brain development [95–97]. However, although the anterior visceral endoderm expressing *Hhex* is involved in brain development, these cells do not contribute directly to the developing brain [97]. They instead serve as a signaling center, which induces the patterning of the anterior neural plate. Furthermore, *Hhex* is expressed in the liver, thyroid and endothelial precursor cells [97]. The differences in *Hhex* expression patterns between *E. rowelli*, *D. melanogaster* and vertebrates indicate that this gene might play different roles in these taxa, so that its ancestral function remains unresolved.

Neural expression of *Emx* only occurs in late developmental stages of *E. rowelli* in a continuous, non-segmental pattern in the brain and the ventral nerve cords. This is in line with previous studies of the onychophoran nervous system, which revealed a medullary rather than ganglionic structure of the ventral nerve cords, with somata distributed along each nerve cord ([54, 56, 57, 60, 77]; but see [87] for an opposing view). Similarly, neural expression of *Emx* has also been reported from various other arthropods, including chelicterates and hexapods, indicating a possible conserved function of this gene in nervous system development [36, 38]. In contrast to the non-segmental neural *Emx* expression in onychophorans, however, arthropods show a segmental expression of *Emx* in subsets of neuronal precursors [35, 36, 38, 99]. In the chelicerates *Euscorpius flavicaudis* and *Tegenaria saeva*, early expression of *Emx* appears in the pre-cheliceral segment that gives rise to parts of the brain [36]. This expression is followed by a segmental expression in clusters of neural precursors along the ventromedial neuroectoderm [36]. This is similar to *Emx* expression in neural precursors of *T. castaneum* and late segmental expression in neural precursors in *D. melanogaster* [50, 99]. Furthermore, *Emx* is involved in early aspects of arthropod neurogenesis [35–38, 99], whereas its neural expression is restricted to late developmental stages in onychophorans. These divergent expression patterns suggest considerable differences in neural development between onychophorans and arthropods, although neural expression of *Emx per se* might have already existed in the panarthropod or even metazoan ancestor. This follows from observations in other metazoans, including acoels [14], cnidarians [100], spiralians [101], and vertebrates [101–107]. In the cnidarian *Acropora millepora*, for example, *Emx* is expressed in neurons that are located in the aboral half of the planula larva [100]. In hatchlings of the acoel *Convolutriloba longifissura*, *Emx* is expressed primarily along the ventral side of the nervous system [14]. In the annelid *P. dumerilii* and various vertebrates, *Emx* is involved in the development of different structures of the brain [101–107]. These similarities suggest a conserved role of *Emx* in neural development in the last common ancestor of bilaterians.

In contrast to the broad expression of *BarH*, *Hhex*, and *Emx* in neural tissue of the onychophoran *E. rowelli*, the expression of *Nedx* and *Bari* is confined to distinct domains in the developing brain, including its central neuropil and the antennal tracts, but is clearly absent from the developing nerve cords. To our knowledge, neural expression of *Nedx* has not been reported from any other animal so far. Similarly, preliminary expression data of *Bari* are so far only available from *D. melanogaster* using high-throughput screening conducted by the Berkeley *Drosophila* Genome Project [91–94]. These data revealed a faint, ubiquitous expression throughout development. Since the corresponding data are missing from other bilaterians, the ancestral role of *Bari* in animal development remains unresolved.

### Conserved involvement of *Nedx* in muscle development of onychophorans and arthropods

Prior to the expression of *Nedx* in tissues associated with the developing brain, this gene is expressed in distinct segmental, mesodermal domains dorsal to each limb bud in embryos of *E. rowelli*. In later developmental stages, these domains extend distally into each limb as well as dorsally towards the rim of embryonic and extra-embryonic tissue. To our knowledge, the expression of *Nedx* has so far only been reported from the fruit fly *D. melanogaster*, where its homolog *lateral muscles scarcer* (*lms*) is involved in both embryonic and adult muscle formation [108]. During embryonic development, *lms* serves as a typical muscle identity gene responsible for the formation of the lateral muscles LT1–LT4 [108]. In the larvae, *lms* is expressed in subsets of adepithelial cells of the wing and leg imaginal discs. While its expression in the leg imaginal discs might be involved in the formation of muscles that extend from the thorax into the coxae, the expression in the wing imaginal discs is involved in the development of direct flight musculature, which is attached to the internal projections of the wing hinge and is essential for the fine control during the flight, such as wing positioning and steering [108, 109].

The position and fibrous appearance of expression in each limb bud of *E. rowelli* indicate that this pattern corresponds to a specific muscle within the onychophoran limb. Interestingly, the *Nedx* domain extends into the dorsal trunk, indicating that this gene might specify a limb muscle with an extrinsic portion, which attaches to the dorsolateral body wall. However, since development of the somatic musculature of onychophorans has not been studied yet in detail and since the exact number and arrangement of individual leg muscles is controversial [110–115], the relation of this expression pattern to the development of a particular muscle or set of muscles remains unclear. Nevertheless, the similarities between *E. rowelli* and *D. melanogaster* indicate that *Nedx* might have been involved in the development and specification of specific limb muscles in the last common ancestor of onychophorans and arthropods.

### *BarH* expression in the developing limbs of *E. rowelli* does not support a conserved role in distal limb development in panarthropods

In addition to the expression in the brain, nerve cords and external sensory structures of *E. rowelli*, *BarH* is expressed in a diffuse pattern in the mesoderm of the developing limbs from an early stage onwards. In addition to this diffuse pattern, a prominent stripe-like domain appears in the developing limbs later in development. In *D. melanogaster*, *BarH* expression occurs in the larval leg discs, where it is responsible for the development of the fourth and fifth tarsal segments as well as the establishment of the sharp tarsal/pretarsal boundary [34]. Together with other transcription factors encoded by *apterous*, *aristaless*, *spineless* and *lim1*, *BarH* comprises a gene regulatory network for the development of the distal-most portion of each leg [34, 116, 117]. It has been proposed that this gene regulatory network might be an ancestral feature of arthropods and that their distal limb portion represents part of the ancestral ground state of limb morphology that predates the arthropods [117].

If so, one would expect a similar pattern of expression in the lobopods of onychophorans – unjointed, tube-like limbs that are also found in tardigrades (water bears) (e.g., refs [118, 119]) and Cambrian lobopodians (e.g. ref. [120]) and, thus, were most likely present in the last common ancestor of panarthropods [54]. However, our data show considerable differences in the expression pattern of *BarH* compared to that in *D. melanogaster*. Instead of being restricted to the distal ectoderm, this gene is expressed along the entire proximo-distal extent of limb mesoderm in *E. rowelli*, and it is absent from the ectoderm, indicating that this gene might be involved in mesoderm differentiation and muscle development rather than distal limb patterning in onychophorans. This is in line with the expression data of other genes involved in the distal patterning cascade, including *aristaless*, *spineless*, *clawless*, *zinc finger homeodomain 2*, *rotund* and *Lim1*, which show a number of conserved and derived features in onychophorans [88]. Thus, if these genes are part of an ancestral patterning mechanism, one would have to assume that this pattern was modified either in onychophorans or in the lineage leading to hexapods. Otherwise, distal limb patterning would have evolved independently in onychophorans and arthropods [88]. As pointed out by Oliveira et al. [88], the lack of comparative data from non-insect arthropods and basally branching insects hampers clear evolutionary conclusions.

### No evidence for a head gap gene-like expression of *Emx* in the onychophoran embryo

Gene expression analyses of the *Emx* ortholog *empty spiracles* (*ems*) in the fruit fly *D. melanogaster* revealed an involvement of this gene in early head formation [49, 50]. Together with *orthodenticle* (*otd*) and *buttonhead* (*btd*), it is expressed in a stereotypic pattern of overlapping domains in the developing head, similar to the expression of trunk gap genes, which is why they were referred to as the head gap genes [35, 36, 38, 121, 122]. Similar gap gene-like expression patterns of *otd* and *btd* have been shown to occur in the myriapod *G. marginata*, suggesting a conserved role of these genes in early head patterning in myriapods and hexapods [123]. However, recent gene expression studies in other hexapods, including *T. castaneum* [38], *Oncopeltus fasciatus* [37] and *Apis mellifera* [35], as well as in the chelicerates *Euscorpius flavicaudis* and *Tegenaria saeva* [36], and the myriapod *S. maritima* [124] have shown that many of these genes do not exhibit the typical head gap gene-like patterning. In other arthropods, *Emx* is instead expressed in the ventrolateral ectoderm in a segmentally reiterated pattern along the anterior border of each segment [35–38]. Interestingly, a similar segmental pattern does indeed also occur in embryos of *D. melanogaster* with a temporal delay separating the early gap gene-like expression from the subsequent patterning of the trunk, suggesting that the patterns observed in other hexapods and chelicerates rather correspond to this late *Emx* expression in *D. melanogaster* [36, 49, 50, 99].

Similarly, even though *Emx* is first expressed in a single domain in the anterior portion of the jaw segment in *E. rowelli*, this gene does not show any characteristics of a head gap gene. Instead, similar domains appear in the subsequent segments shortly after this initial expression in the jaw segment, thus following the anterior-to-posterior progression in embryonic development [125, 126]. These domains are restricted to the lateral ectoderm early in development and ventrally in later developmental stages. A similar expression pattern of *Emx* has been reported from the onychophoran *Euperipatoides kanangrensis* [127], which is closely related to *E. rowelli*. This is strikingly similar to the situation in arthropods, in which the segmental *Emx* pattern occupies a lateral position in the anterior portion of each segment [35–38, 49, 50, 99]. Thus, our data as well as the previously published data from *E. kanangrensis* [127] support the hypothesis that *Emx* was expressed in a segmentally reiterated pattern in the ventrolateral ectoderm rather than functioned as a head gap gene in the last common ancestor of onychophorans and arthropods, and that the head gap gene-like expression might have evolved in the Manibulata and was lost in the lineages including the myriapod *S. maritima*, and the hexapods *T. castaneum*, *Oncopeltus fasciatus* and *Apis mellifera*. Alternatively, the head gap gene-like expression might have evolved independently in the myriapod subclade, which includes *G. marginata*, and the insect subclade (long germ band insects?), which includes *D. melanogaster* [35–38, 123].

## Conclusions

Our analysis of the NKL genes in embryos of the onychophoran *E. rowelli* revealed that they are involved in various developmental processes. The major findings of this study can be summarized as follows:

- *NK2.1* and *vax* are expressed in overlapping domains in the ventromedial ectoderm of the cephalic lobes as well as in tissue surrounding the cerebral grooves, which likely give rise to neurosecretory cells in the ventromedial protocerebrum and the hypocerebral organs, respectively. These patterns are reminiscent of what has been described from other nephrozoans, indicating that *NK2.1* and *vax* are involved in a conserved molecular patterning system regulating the formation of neurosecretory cells that might have already existed in the last common ancestor of Nephrozoa.
- *BarH* is expressed in the central nervous system of onychophorans, which resembles its expression in the nervous system of other bilaterians, indicating that this gene might have been involved in patterning the central nervous system in the last common ancestor of bilaterians or at least nephrozoans. Furthermore, *BarH* is expressed in external sensory organs in both onychophorans and arthropods, suggesting that these organs share a common origin.
- The neural expression of *Emx* in onychophorans, arthropods and various other bilaterian taxa indicates a shared function of *Emx* in neural development, which might have been inherited from the last common ancestor of Bilateria. In contrast to this, the segmental rather than head gap gene-like expression of *Emx* in onychophorans supports the hypothesis that the latter expression is a derived feature of either the Mandibulata or the myriapod and insect subgroups including *G. marginata* and *D. melanogaster*, respectively.
- *Hhex*, *Nedx* and *Bari* are expressed in distinct regions of the nervous system, including the brain and the ventral nerve cords. However, due to major differences in expression patterns between *E. rowelli*, *D. melanogaster* and vertebrates as well as the lack of comparative data from other bilaterians, the ancestral patterns and putative functions of these genes remain unresolved.
- *Nedx* and *BarH* are expressed in distinct mesodermal regions of the developing limbs in *E. rowelli*. While *Nedx* might be involved in the development of limb musculature in both onychophorans and arthropods, considerable differences of *BarH* expression between onychophorans and arthropods indicate divergent roles of this gene in limb formation.

## Methods

### Specimen collection and sample preparation

Female specimens of *Euperipatoides rowelli* [128] (Peripatopsidae) were collected in the Tallaganda State Forest (New South Wales, Australia; 35°26′S, 149°33′E, 954 m) in October 2011, January 2013 and October 2016. Permissions for collection and export of specimens were obtained from the National Parks & Wildlife Service New South Wales (permit numbers: SL100159, SPPR0008 and SL101720) and the Department of Sustainability, Environment, Water, Population and Communities (permit numbers: PWSP104061, PWSP208163, and PWS2016-AU-001023), respectively. The animals were maintained in the laboratory following the instructions described in ref. [129]. Embryos were dissected from female genital tracts, staged and stored as described previously [16, 126, 130–132].

### Identification of NKL homologs, phylogenetic analysis, and nomenclature

Library preparation, assembly of the embryonic transcriptomes from *E. rowelli*, and identification of putative NKL gene homologs was performed as described previously [27, 133, 134]. The sequences of all identified NKL homeobox genes were made available in GenBank (see Table 2 for accession numbers). Since the current nomenclature of the NK genes is confusing due to the existence of different synonyms for each gene in different taxa, we use the general gene family names to avoid further confusion (Table 2; see also Table 1 in ref. [5], Table 2 in ref. [9] and Table 1 in ref. [25] for further information on NK gene families and their synonyms).

**Table 2:** GenBank accession numbers of the identified NKL genes from *E. rowelli*, and commonly used synonyms for each gene

Gene name	Accession number	Synonyms
*NK2.1*	MH971984	*nkx2.1*, *nkx2–1*, *ceh-24*, *ceh-27*, *Scarecrow* (*Scro*), *scarecrow* (*scro*), *nkx2–4*
*vax*	MH971979	*ceh-5*, *ceh-7*
*Emx*	MH971980	*E5*, *empty spiracles* (*ems*), *ceh-2*, *ceh-23*
*Nedx*	MH971978	*CG13424*, *lateral muscles scarcer* (*lms*)
*Hhex*	MH971983	*Hex*, *PRHX*, *HOX11L-PEN*, *CG7056*, *pha-2*
*Bari*	MH971981	*CG11085*
*BarH*	MH971982	*BarHL*, *B-H1 and 2*, *ceh-30*, *ceh-31*

### RNA isolation, amplification, molecular cloning, and whole-mount *in situ* hybridization

RNA isolation and cDNA synthesis were performed as described previously [16, 126]. Amplification of fragments of the NKL genes *NK2.1*, *Emx*, *BarH, Bari*, *Hhex*, *Nedx* and *vax* was performed using specific primers (Table 3). The fragments were cloned as described previously [27]. Probe preparation and whole-mount *in situ* hybridization was performed as described previously [27, 130] with the modifications described in ref. [27]. After *in situ* hybridization, the embryos were counterstained with the DNA-selective fluorescent dye 4′,6-Diamidin-2-phenylindol (DAPI; Thermo Scientific, formerly Invitrogen; diluted 1:1000 in 0.1 M PBS, pH 7.4: 5 mg/ml NaCl, 14 mg/ml Na_2_HPO_4_-2H_2_O, 3.2 mg/ml NaH_2_PO_4_-H_2_O) for 1 h and stored in 70% glycerol. Cross sections were prepared manually using a razor blade. Controls with the sense probes of each gene were performed using same protocol as described above. Early to intermediate embryonic stages (0 to IV) did not show any labeling. Staining artifacts appearing in body cavities and cuticular structures of late developmental stages [135, 136] were absent in the controls treated with chitinase/chymotrypsin prior to hybridization, except for unspecific labeling in the cavities of the developing antennae, and the sclerotized jaws and claws of stage VII embryos (Additional file 3). We therefore conclude that the staining obtained with the antisense probes is specific for all genes studied.

**Table 3:** Specific primers used to amplify the transcripts of NKL genes from cDNA of the onychophoran *E. rowelli*

Gene	Fragment length (in bases)	Direction	Primer sequence	Annealing Temperature (°C)
*NK2.1*	823	Forward	TTCTGCATAATAAGGCGGGC	62
		Reverse	TTACCACTCCCGTCATTTCC	
*vax*	543	Forward	TATCGACTGTCTCCTCTGTCC	62
		Reverse	AAAGACGGCCTTCGAAATGGC	
*Emx*	742	Forward	CCACGCCTATAGTTCCTAAGC	62
		Reverse	TCCTCACCTTGAAGTGTCTCC	
*Nedx*	795	Forward	ATGGAAGAAGAGGCCACCAG	62
		Reverse	AGGAGGAATGACACCCATAGG	
*Hhex*	696	Forward	CATTGGGATGGCTACCTATGG	62
		Reverse	CCTGTACTGCTAGCAACAACC	
*Bari*	411	Forward	CAAACTACTAAATCAAAGAAACCGAGA	62
		Reverse	TTAGTGTTGACACCCAGGTACAAACAT	
*BarH*	680	Forward	GGAGGGTTCTAGCAGTAATGC	58
		Reverse	GTGTTGGATAGGGCCAATAGG	

### Microscopy and image processing

The embryos were analyzed and imaged using the stereomicroscope Axio Zoom V16 (Carl Zeiss MicroImaging GmbH, Jena, Germany) equipped with an Axiocam 503 color digital camera (Carl Zeiss MicroImaging GmbH). Light micrographs, and superimposed light and DAPI micrographs were taken at different focal planes which were then merged to a single projection using the ZEN 2012 blue edition software version 1.1.2.0 (Carl Zeiss MicroImaging GmbH). All micrographs were adjusted for color balance, brightness and contrast using the ZEN 2012 blue edition software and Adobe (San Jose, CA, USA) Photoshop CS 5.1. Final panels and diagrams were designed using Adobe Illustrator CS 5.1.

## Additional files

Additional file 1: NKL gene complements of different bilaterian species. Blue and grey background indicates presence and absence of NKL genes, respectively. Numbers indicate the number of genes, dashes indicate the absence thereof, numbers in brackets indicate the number of pseudogenes, question marks indicate missing data. The gene complements were retrieved from publicly available data as well as the sources specified below the table. (TIF 9969 kb)

Additional file 2: Expression of *Nedx* in embryos of the onychophoran *E. rowelli*. Anterior is left and dorsal is up in all images; developing trunk segments are numbered. **A** Stage III embryo in lateral view. **B** Stage IV embryo in lateral view. Inset shows detail of a cross section of the same embryo. **C** Late stage IV embryo in lateral view. Abbreviations: at, developing antenna; cl, cephalic lobe; de, dorsal extra-embryonic tissue; jw, developing jaw; po, proctodeum; sp.; developing slime papilla; ve, ventral extra-embryonic tissue. Scale bars: 500 μm. (DOCX 44 kb)

Additional file 3: Controls using the sense probes of all investigated genes in late developmental stages of the onychophoran *E. rowelli*. Anterior is left in all images. Images on the left show undigested embryos, images on the right embryos that were treated with chymotrypsin/chitinase prior to hybridization. Note that undigested embryos show large amounts of unspecific signal in the developing cuticle, the developing ventral and preventral organs and sclerotized jaws and claws. Scale bars: 500 μm. (TIF 6059 kb)
