# Supplementary figures and images for: Expression of NK genes that are not part of the NK cluster in the onychophoran Euperipatoides rowelli (Peripatopsidae)
Source: BMC Dev Biol. 2019 Apr 15;19:7. doi: 10.1186/s12861-019-0185-9 (PMC6466738; doi:10.1186/s12861-019-0185-9)

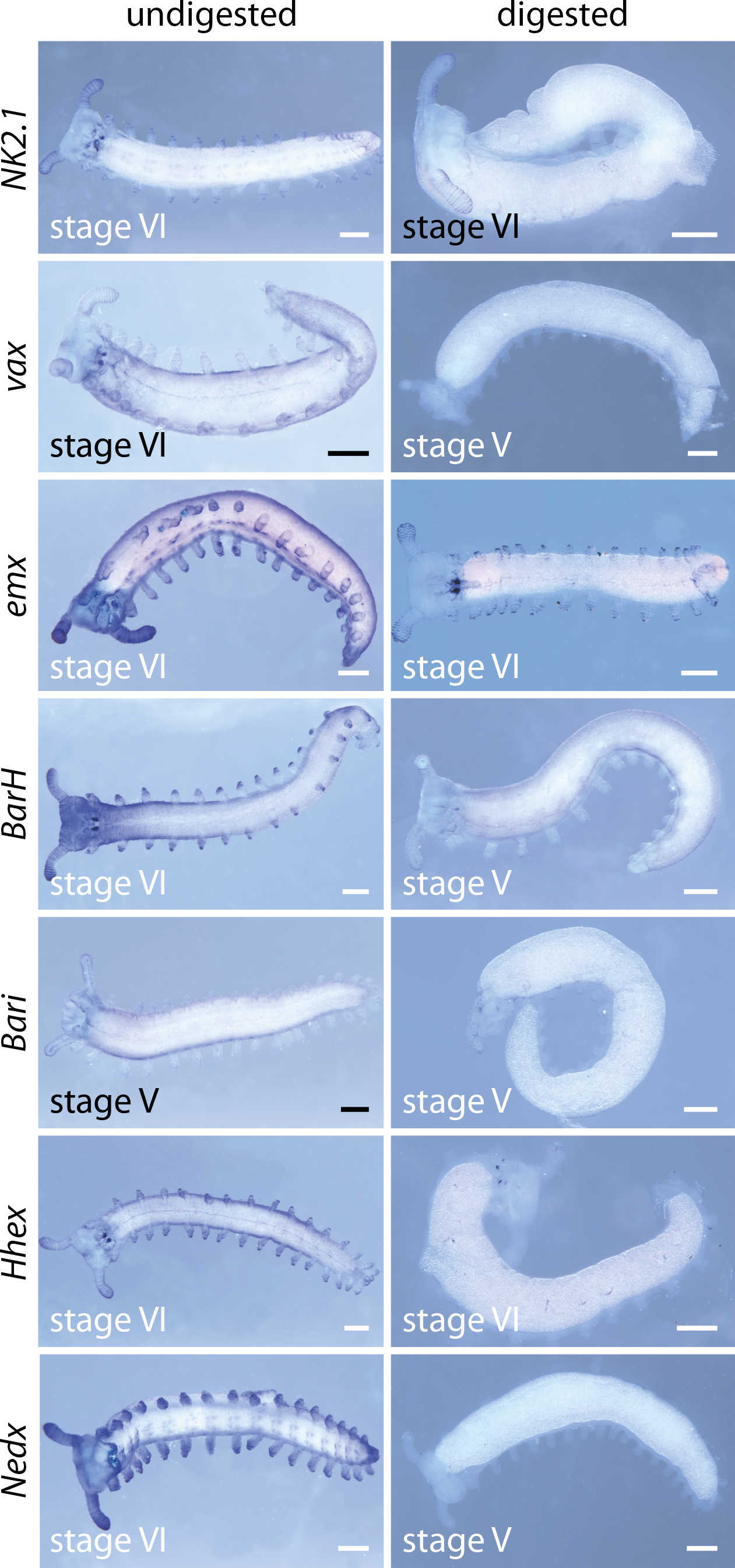

Supplement: Supplementary file 1 — NKL gene complements of different bilaterian species. Blue and grey background indicates presence and absence of NKL genes, respectively. Numbers indicate the number of genes, dashes indicate the absence thereof, numbers in brackets indicate the number of pseudogenes, question marks indicate missing data. The gene complements were retrieved from publicly available data as well as the sources specified below the table. (TIF 9969 kb) [file 12861_2019_185_MOESM1_ESM.tif]

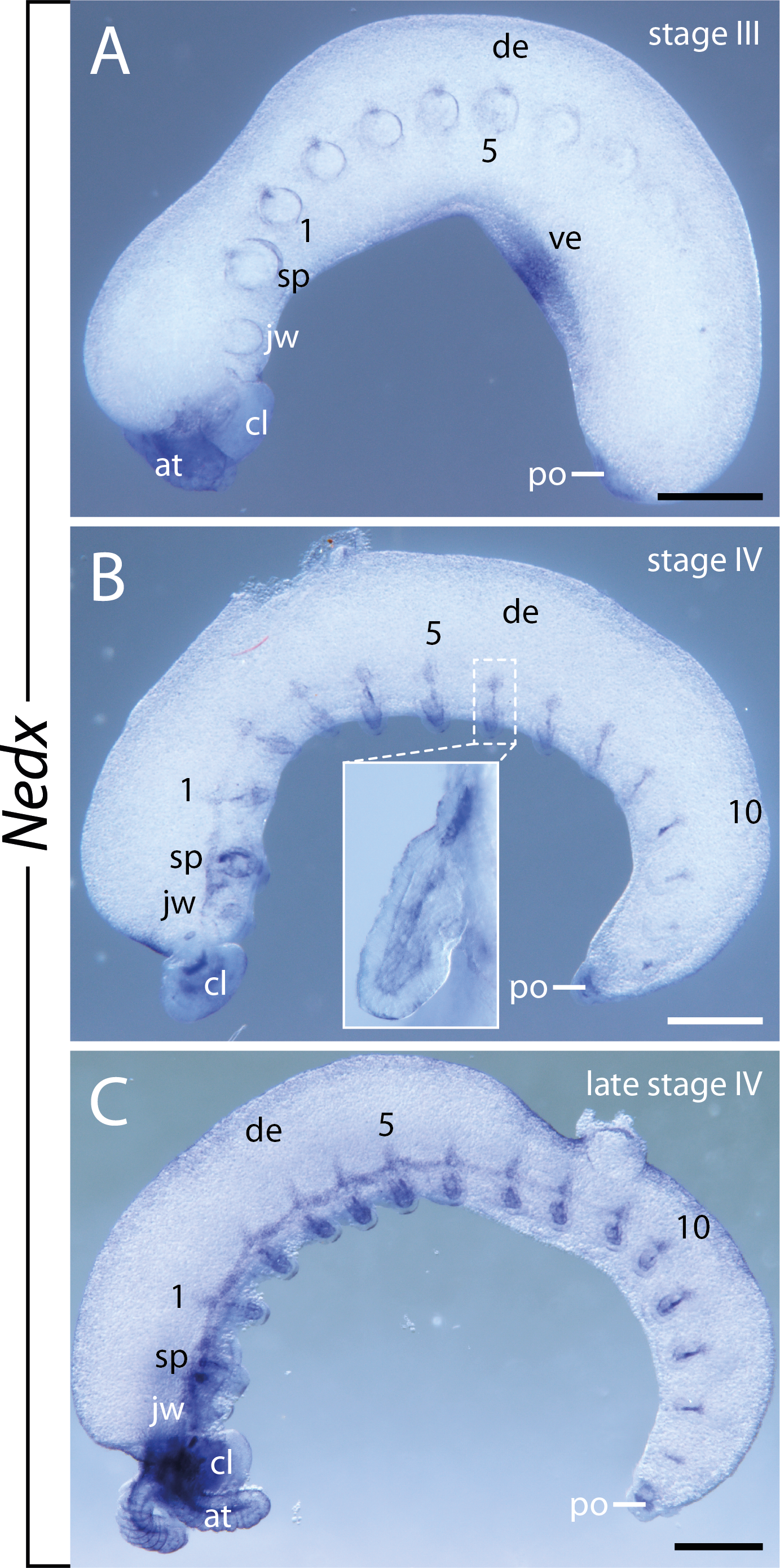

Supplement: Supplementary file 3 — Controls using the sense probes of all investigated genes in late developmental stages of the onychophoran E. rowelli. Anterior is left in all images. Images on the left show undigested embryos, images on the right embryos that were treated with chymotrypsin/chitinase prior to hybridization. Note that undigested embryos show large amounts of unspecific signal in the developing cuticle, the developing ventral and preventral organs and sclerotized jaws and claws. Scale bars: 500 μm. (TIF 6059 kb) [file 12861_2019_185_MOESM3_ESM.tif]
