# Supplementary material for: Expression of NK genes that are not part of the NK cluster in the onychophoran Euperipatoides rowelli (Peripatopsidae)
Source: BMC Dev Biol. 2019 Apr 15;19:7. doi: 10.1186/s12861-019-0185-9 (PMC6466738; doi:10.1186/s12861-019-0185-9)
Supplement: Supplementary file 2 — Expression of Nedx in embryos of the onychophoran E. rowelli. Anterior is left and dorsal is up in all images; developing trunk segments are numbered. A Stage III embryo in lateral view. B Stage IV embryo in lateral view. Inset shows detail of a cross section of the same embryo. C Late stage IV embryo in lateral view. Abbreviations: at, developing antenna; cl, cephalic lobe; de, dorsal extra-embryonic tissue; jw, developing jaw; po, proctodeum; sp.; developing slime papilla; ve, ventral extra-embryonic tissue. Scale bars: 500 μm. (DOCX 44 kb) [file 12861_2019_185_MOESM2_ESM.docx]

**Additional file 2:** NKL gene complements of different bilaterian species. Blue and grey background indicates presence and absence of NKL genes, respectively. Numbers indicate the number of genes, dashes indicate the absence thereof, numbers in brackets indicate the number of pseudogenes, question marks indicate missing data. The gene complements were retrieved from publicly available data as well as the sources specified below the table.

|  | ***Amphimedon queenslandica ^1^*** | ***Nematostella vectensis ^2^*** | ***Lottia gigantean ^3^*** | ***Platynereis dumerillii* ^4^** | ***Caenorhabditis elegans* ^5^** | ***Ramazzottius varieornatus* ^6^** | ***Hypsibius exemplaris* ^7^** | ***Euperipatoides rowelli* ^8^** | ***Parasteatoda tepidariorum* ^9^** | ***Strigamia maritima* ^10^** | ***Daphnia pulex* ^11^** | ***Tribolium castaneum* ^5^** | ***Apis mellifera* ^5^** | ***Drosophila melanogaster* ^5^** | ***Branchiostoma floridae* ^5^** | ***Xenopus tropicalis* ^5^** | ***Gallus gallus* ^5^** | ***Danio rerio* ^5^** | ***Mus musculus* ^5^** | ***Homo sapiens* ^5^** |
| --- | --- | --- | --- | --- | --- | --- | --- | --- | --- | --- | --- | --- | --- | --- | --- | --- | --- | --- | --- | --- |
| ***NK2.1*** | — | ? | 1 | 1 | 1 | 1 | — | 1 | — | 1 | 1 | 1 | 1 | 1 | 1 | 4 | 1 | 3 | 2 | 2 |
| ***NK2.2*** | — | 5^?^ | 2 | 1 | 1 | 1 | — | 1 | 2 | 2 | 1 | 1 | 1 | 1 | 1 | 2 | 1 | 3 | 2 | 2 |
| ***Abox*** | — | — | — | ? | 1 | 1 | 1 | — | — | 1 | — | 1 | 1 | 1 | 1 | — | — | — | — | — |
| ***Ro*** | — | 1 | — | ? | 1 | 1 | 1 | — | 1 | 1 | — | 1 | 1 | 1 | 1 | — | — | — | — | — |
| ***Nedx*** | — | 2 | — | ? | — | 1 | 1 | 1 | — | 1 | — | 1 | 1 | 1 | 2 | — | — | — | — | — |
| ***vax*** | — | 2 | — | ? | 2 | — | — | 1 | — | 1 | — | — | — | — | 1 | 2 | 1 | 2 | 2 | 2 |
| ***Noto*** | — | 1 | — | ? | — | — | — | — | — | 1 | 1 | 1 | 1 | 1 | 1 | 1 | 2 | 1 | 1 | 1 |
| ***Emx*** | — | 2 | 2 | ? | 2 | 1 | 1 | 1 | 4 | 1 | 1 | 1 | 1 | 2 | 3 | 4 | 2 | 3 | 2 | 2 |
| ***BarH*** | 1 | — | 2 | ? | 2 | 2 | 3 | 1 | 2 | — | 2 | 1 | 1 | 2 | 1 | 2 | — | 3 | 2 | 2 |
| ***Bari*** | — | — | — | ? | — | — | — | 1 | 1 | 1 | 1 | 1 | 1 | 1 | 1 | — | — | — | — | — |
| ***Barx*** | — | — | — | ? | — | — | 2 | — | 1 | — | — | — | — | — | 1 | 2 | 1 | 2 | 2 | 2 |
| ***Hlx*** | — | 7 | — | ? | — | — | — | — | — | — | — | 1 | 1 | 1 | 1 | 1 | — | 1 | 1 | 1 |
| ***Dbx*** | — | — | — | ? | — | — | — | — | — | — | — | 1 | 1 | 1 | 1 | 2 | 2 | 3 | 2 | 2 |
| ***Bsx*** | — | — | — | ? | 1 | — | — | — | — | — | — | 1 | 1 | 1 | 1 | 1 | 1 | 1 | 1 | 1 |
| ***Hhex*** | 1 | 1 | 1 | ? | 1 | 1 | 1 | 1 | — | 1 | — | 1 | — | 1 | 1 | 2 | 1 | 1 | 1 | 1 |
| ***Nanog*** | — | — | — | ? | — | — | — | — | — | — | — | — | — | — | — | — | — | — | 3(2) | 12 (11) |
| ***Ventx*** | — | — | — | ? | — | — | — | — | — | — | — | — | — | — | 2 | 6 | 1 | 1 | — | 8 (7) |

^1^ [[1](#_ENREF_1)]; ^2^ [[2](#_ENREF_2)]; ^3^ [[3](#_ENREF_3)]; ^4^ [[4](#_ENREF_4), [5](#_ENREF_5)]; ^5^ [[6](#_ENREF_6), [7](#_ENREF_7)]; ^6^ [[8](#_ENREF_8)]; ^7^ [[9-11](#_ENREF_9)]; ^8^ [[12](#_ENREF_12)]; ^9^ [[13](#_ENREF_13)]; ^10^ [[14](#_ENREF_14)]; ^11^ [[15](#_ENREF_15)]

1. Fahey B, Larroux C, Woodcroft BJ, Degnan BM: **Does the high gene density in the sponge NK homeobox gene cluster reflect limited regulatory capacity?** *The Biological Bulletin* 2008, **214:**205–217.

2. Ryan JF, Burton PM, Mazza ME, Kwong GK, Mullikin JC, Finnerty JR: **The cnidarian-bilaterian ancestor possessed at least 56 homeoboxes: evidence from the starlet sea anemone, *Nematostella vectensis*.** *Genome Biol* 2006, **7:**R64–R64.

3. Simakov O, Marletaz F, Cho S-J, Edsinger-Gonzales E, Havlak P, Hellsten U, Kuo D-H, Larsson T, Lv J, Arendt D, et al: **Insights into bilaterian evolution from three spiralian genomes.** *Nature* 2013, **493:**526–531.

4. Saudemont A, Dray N, Hudry B, Le Gouar M, Vervoort M, Balavoine G: **Complementary striped expression patterns of NK homeobox genes during segment formation in the annelid *Platynereis*.** *Dev Biol* 2008, **317:**430–443.

5. Denes AS, Jékely G, Steinmetz PRH, Raible F, Snyman H, Prud'homme B, Ferrier DEK, Balavoine G, Arendt D: **Molecular architecture of annelid nerve cord supports common origin of nervous system centralization in Bilateria.** *Cell* 2007, **129:**277–288.

6. Zhong Y-f, Holland PWH: **HomeoDB2: functional expansion of a comparative homeobox gene database for evolutionary developmental biology.** *Evol Dev* 2011, **13:**567–568.

7. Zhong Y-F, Butts T, Holland PWH: **HomeoDB: a database of homeobox gene diversity.** *Evol Dev* 2008, **10:**516–518.

8. Hashimoto T, Horikawa DD, Saito Y, Kuwahara H, Kozuka-Hata H, Shin-I T, Minakuchi Y, Ohishi K, Motoyama A, Aizu T, et al: **Extremotolerant tardigrade genome and improved radiotolerance of human cultured cells by tardigrade-unique protein.** *Nat Commun* 2016, **7:**12808.

9. Hering L, Henze MJ, Kohler M, Bleidorn C, Leschke M, Nickel B, Meyer M, Kircher M, Sunnucks P, Mayer G: **Opsins in Onychophora (velvet worms) suggest a single origin and subsequent diversification of visual pigments in arthropods.** *Mol Biol Evol* 2012, **29**.

10. Hering L, Mayer G: **Analysis of the Opsin repertoire in the tardigrade *Hypsibius dujardini* provides insights into the evolution of Opsin genes in Panarthropoda.** *Genome Biol Evol* 2014, **6:**2380–2391.

11. Arakawa K, Yoshida Y, Tomita M: **Genome sequencing of a single tardigrade *Hypsibius dujardini* individual.** *Sci Data* 2016, **3:**160063.

12. Treffkorn S, Kahnke L, Hering L, Mayer G: **Expression of NK cluster genes in the onychophoran *Euperipatoides rowelli*: Implications for the evolution of NK family genes in nephrozoans.** *EvoDevo* accepted.

13. Schwager EE, Sharma PP, Clarke T, Leite DJ, Wierschin T, Pechmann M, Akiyama-Oda Y, Esposito L, Bechsgaard J, Bilde T, et al: **The house spider genome reveals an ancient whole-genome duplication during arachnid evolution.** *BMC Biol* 2017, **15:**62.

14. Chipman AD, Ferrier DEK, Brena C, Qu J, Hughes DST, Schröder R, Torres-Oliva M, Znassi N, Jiang H, Almeida FC, et al: **The first myriapod genome sequence reveals conservative arthropod gene content and genome organisation in the centipede *Strigamia maritima*.** *PLoS Biol* 2014, **12:**e1002005.

15. Colbourne JK, Pfrender ME, Gilbert D, Thomas WK, Tucker A, Oakley TH, Tokishita S, Aerts A, Arnold GJ, Basu MK, et al: **The Ecoresponsive Genome of *Daphnia pulex*.** *Science (New York, NY)* 2011, **331:**555–561.
